# Evolutionarily conserved partial gene duplication in the Triticeae tribe of grasses confers pathogen resistance

**DOI:** 10.1186/s13059-018-1472-7

**Published:** 2018-08-15

**Authors:** Jeyaraman Rajaraman, Dimitar Douchkov, Stefanie Lück, Götz Hensel, Daniela Nowara, Maria Pogoda, Twan Rutten, Tobias Meitzel, Jonathan Brassac, Caroline Höfle, Ralph Hückelhoven, Jörn Klinkenberg, Marco Trujillo, Eva Bauer, Thomas Schmutzer, Axel Himmelbach, Martin Mascher, Barbara Lazzari, Nils Stein, Jochen Kumlehn, Patrick Schweizer

**Affiliations:** 1 Leibniz Institut für Pflanzengenetik und Kulturpflanzenforschung (IPK Gatersleben), Corrensstrasse 3, D-06466 Stadt Seeland, Germany; 2 Technische Universität München, Emil-Ramann-Straße 2, D-85354 Freising, Germany; 3 Leibniz Institut für Pflanzenbiochemie, Weinberg 3, D-06120 Halle (Saale), Germany; 4 Technische Universität München, Liesel-Beckmann-Straße 2, D-85354 Freising, Germany; 5 Parco Technologico Padano, Via Einstein, Loc. Cascina Codazza, 26900 Lodi, Italy; 6 Albert-Ludwigs-Universität Freiburg, Institut für Biologie II, Zellbiologie, D-79104 Freiburg, Germany

**Keywords:** Partial gene duplication, Neo-functionalization, Disease resistance, Triticeae grasses

## Abstract

**Background:**

The large and highly repetitive genomes of the cultivated species *Hordeum vulgare* (barley), *Triticum aestivum* (wheat), and *Secale cereale* (rye) belonging to the Triticeae tribe of grasses appear to be particularly rich in gene-like sequences including partial duplicates. Most of them have been classified as putative pseudogenes. In this study we employ transient and stable gene silencing- and over-expression systems in barley to study the function of *HvARM1* (for *H. vulgare* Armadillo 1), a partial gene duplicate of the U-box/armadillo-repeat E3 ligase *HvPUB15* (for *H. vulgare* Plant U-Box 15).

**Results:**

The partial *ARM1* gene is derived from a gene-duplication event in a common ancestor of the Triticeae and contributes to quantitative host as well as nonhost resistance to the biotrophic powdery mildew fungus *Blumeria graminis*. In barley, allelic variants of *HvARM1* but not of *HvPUB15* are significantly associated with levels of powdery mildew infection. Both HvPUB15 and HvARM1 proteins interact in yeast and plant cells with the susceptibility-related, plastid-localized barley homologs of THF1 (for Thylakoid formation 1) and of ClpS1 (for Clp-protease adaptor S1) of *Arabidopsis thaliana*. A genome-wide scan for partial gene duplicates reveals further events in barley resulting in stress-regulated, potentially neo-functionalized, genes.

**Conclusion:**

The results suggest neo-functionalization of the partial gene copy *HvARM1* increases resistance against powdery mildew infection. It further links plastid function with susceptibility to biotrophic pathogen attack. These findings shed new light on a novel mechanism to employ partial duplication of protein-protein interaction domains to facilitate the expansion of immune signaling networks.

**Electronic supplementary material:**

The online version of this article (10.1186/s13059-018-1472-7) contains supplementary material, which is available to authorized users.

## Background

Plants respond to pathogen attack by the activation of their innate immunity system, which is triggered by the perception of pathogen-associated molecular patterns (PAMPs) via pattern recognition receptors [1]. Successful plant pathogens manipulate their hosts by complex arsenals of secreted effector proteins, which suppress immunity and co-opt cellular host functions for accommodation and nutritional exploitation [2]. Nonhost plant species, on the other hand, exhibit nonhost resistance (NHR), which protects them from the vast majority of attacks by pathogens that have adapted to different, more or less closely related, plant species [3]. The outcome of pathogen-host interactions can vary from immune to highly susceptible, depending on the presence or absence of major resistance genes or on different levels of quantitative host resistance (QR). QR is usually determined by several quantitative trait loci (QTL) and may be partially explained by a manifestation of PAMP-triggered immunity (PTI). In contrast to effector-triggered immunity (ETI), it does not confer complete protection but may be more durable in the field [4–6].

All forms of host resistance are temporary results of the co-evolutionary arms race between host plants and their adapted pathogens. As such, pathogens evolve quickly and put enormous selection pressure on host genomes to keep pace with changing virulences [7–10], thereby resulting in strong selective pressure on new resistance and defense genes or gene variants. Besides alternative splicing, gene duplication is an efficient way to create novelty in genomes and is routinely observed for ETI-mediating nucleotide binding domain-leucine rich repeat domain (NB-LRR)-type major resistance genes as well as receptor-like kinases in pairs or clusters of tandem duplicates [11, 12]. Genes can be duplicated as complete or partial copies. In humans, partially duplicated genes have been recognized as a major cause of disease including different forms of cancer [13–15]. The possible contribution of partial gene duplicates to positive traits such as disease resistance is much less examined in animal genetics and apparently unexplored in plants [16–18]. The occurrence of partial gene copies is particularly relevant to the large and highly repetitive genomes of the Triticeae tribe of grasses including *Hordeum vulgare* ssp. *vulgare* (cultivated barley), *Triticum aestivum* (bread wheat), and *Secale cereale* (rye), which were described to be particularly rich in gene-like sequences including partial duplicates, most of which were classified as putative pseudogenes [19–21].

Cultivated barley is nonhost to the non-adapted wheat powdery mildew fungus *Blumeria graminis* f.sp. *tritici* (*Bgt*) but a host of the powdery mildew fungus *B. graminis* f.sp. *hordei* (*Bgh*), which causes up to 30% yield loss in the absence of genetic or chemical control of the disease [22, 23]. The epidemic spread of *B. graminis* is caused by the asexual propagation of the fungus, with a generation time of 5–7 days and massive production of conidiospores (Additional file 1: Figure S1). The interaction between different barley genotypes and *Bgh* isolates represents a well-studied model system for a fungal disease caused by an obligate biotrophic pathogen, and a growing number of host-response factors for defense or disease establishment have been identified [24–26]. The genome of *Bgh* was found to encode more than 500 candidate secreted effector proteins [27]. In several pathogens, a growing number of effectors were found to target components of the plant ubiquitination machinery including plant U-box E3 ligases (PUBs) [28–34]. The covalent attachment of single ubiquitin moieties or polyubiquitin chains to lysine residues of eukaryotic protein substrates can have diverse effects on their fate. Ubiquitination most commonly results in the recognition and degradation of tagged proteins by the 26S proteasome, but it also mediates endosomal sorting into cellular compartments such as the lysosome or the plant vacuole, or contributes to DNA damage responses [35, 36]. The substrate specificity during ubiquitination is determined by the E3 ubiquitin ligases which can be subdivided into three categories, namely HECT, RING/U-box type, and cullin-RING ligases. These proteins mediate ubiquitin ligation in concert with the highly conserved ubiquitin-activating enzyme (E1) and ubiquitin-conjugating enzymes (E2). Due to their central cellular function, components of the ubiquitination system represent central cellular hubs of protein regulation involved in all aspects of plant life. As such, beneficial or parasitic organisms may utilize the ubiquitination machinery [32] to establish susceptible interactions. On the other hand, higher plants exploit ubiquitin-mediated degradation of negative protein regulators of stress-hormone signaling for the initiation of defense responses [36–38].

In a phenotype-driven, transient RNA interference (RNAi) screen for the discovery of *Rnr* (for *Required for nonhost resistance*) genes to the non-adapted wheat powdery mildew fungus *B. graminis* f.sp. *tritici* (*Bgt*), we tested more than 631 barley genes, which were mostly associated with up-regulated transcripts in *Bgt*-attacked barley leaf epidermis [39]. Reduced NHR was reflected by an increased percentage of transformed epidermal cells containing *Bgt* haustoria. This revealed 10 final *Rnr* gene candidates that significantly enhanced nonhost susceptibility upon silencing. Here we present an extensive evolutionary, genomics, and molecular functional study of the neo-functionalized partial gene copy *Rnr5* encoding HvARM1 with homology to plant U-box protein 15 (HvPUB15). Besides its possible role in NHR that allowed its discovery in the NHR RNAi screening, functional analysis suggested *Rnr5* as an important factor of QR against the adapted *Bgh* fungus.

## Results

### Origin and evolution of *HvARM1*

**Fig. 1:**
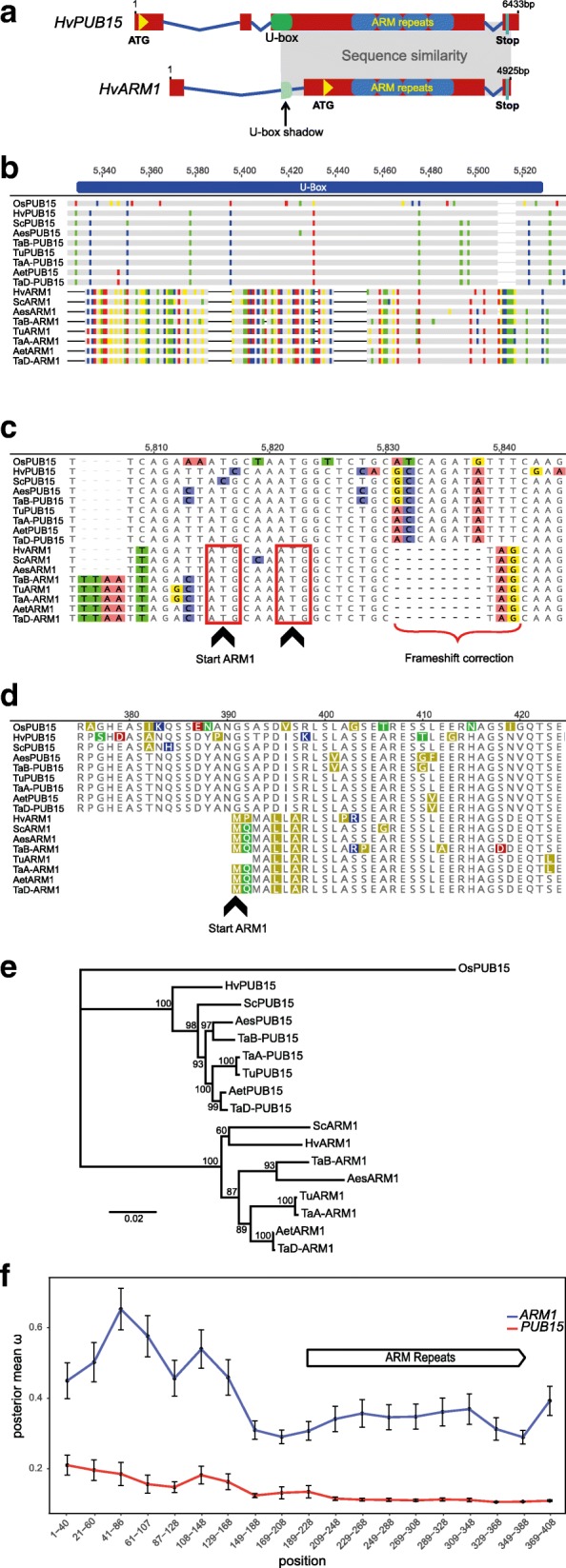
A partial duplication of the E3 ligase gene *PUB15* in Triticeae species gave rise to *ARM1*. **a** Schematic view of the genomic structure of *HvPUB15* and its partial duplicate *HvARM1*. *Red boxes* and *blue lines* represent exon and intron sequences, respectively. The region of high sequence homology is indicated by *light gray shading*. **b** DNA sequence alignment around the U-box domain of PUB15. Note the absence of sequence alignment (gaps and different colors) in the 5′ end of the U-box in ARM1. **c** DNA sequence alignment around the translational start of *ARM1*. The two proposed translation start sites of *ARM1* are inside the *red frames*, and the initial frame of *PUB15* is restored by an 8-bp deletion (frameshift correction) downstream from the first 5 amino acids of *ARM1* and is indicated by a brace. **d** Protein sequence alignment of PUB15 and ARM1 at the N-terminus of ARM1. **e** Phylogenetic tree of both proteins based on alignment of overlapping PUB15 and ARM1 coding sequences. A maximum likelihood tree with *OsPUB15* as outgroup was calculated and bootstrap values (in percent) based on 100 reiterations are indicated along the branches and tree depth (in changes per nucleotide position) by scale bar. **f** Conservative selection at the armadillo-repeat domain of *ARM1* among Triticeae species. The ratio of non-synonymous to synonymous nucleotide exchanges (ω) among *PUB15* and *ARM1* genes overlapping region of six Triticeae species plus rice was calculated. Posterior mean ω + standard error (SE) are reported over 40 non-ambiguous amino acid positions sliding window, with a step size of 20. **a**–**f** Species binomial abbreviations: *Aes*, *Aegilops speltoides*, *Aet*, *Aegilops tauschii* (wild wheat close relatives); *Hv, Hordeum vulgare* (barley); *Os*, *Oryza sativa* (rice); *Sc, Secale cereale* (rye), *Ta, Triticum aestivum* (wheat) followed by homeolog orgin, *Tu*, *Triticum urartu* (wild wheat close relative). **b**–**d** Disagreements to majority consensus are highlighted in *color*. Accession numbers of publicly available sequences are listed in Additional file 1: Table S10

Transient single-cell silencing of *Rnr5* significantly reduced NHR of barley to *Bgt* [39]. BlastX analysis revealed the homology of *Rnr5* to PUBs. PUBs contain an N-terminal U-box domain and a C-terminal armadillo-(ARM) repeat domain [40, 41]. Although *Rnr5* was most closely related to *OsPUB15* in rice [42], it does not appear to be the barley ortholog because the encoded protein of 442 amino acids is considerably shorter than a regular PUB and contains only the C-terminal ARM-repeat region as a conserved domain (Fig. 1a). The barley genome also contains a gene for a full-length PUB protein of 831 amino acids with highest similarity to *OsPUB15* that was therefore named *HvPUB15,* whereas *Rnr5* was designated as *HvARM1* (Table 1). Protein similarity between HvPUB15 and HvARM1 starts at positions L398 and L9 of HvPUB15 and HvARM1, respectively, between the conserved U-box and ARM-repeat regions of HvPUB15 (Additional file 1: Figures S2 and S3a). Sequence similarity between the two genes extends upstream from the *HvARM1* initiating codon spanning the first intron of *HvARM1*, which corresponds to the exon 3 sequence of *HvPUB15,* until it abruptly ends within the U-box sequence of *HvPUB15* (Fig. 1a and b). A further upstream sequence inside *HvARM1* intron 1 as well as the untranslated exon1 sequence did not exhibit significant similarity to any annotated gene or repetitive DNA element in the barley genome. These results strongly suggest that *HvARM1* originated as a partial gene duplicate of *HvPUB15*. An 8-bp deletion downstream from the first five N-terminal amino acids of *HvARM1* restored the initial reading frame (Fig. 1c) because its initiating ATG corresponds to an out-of-frame codon of *HvPUB15* (Fig. 1d). Whole genome shotgun (WGS) sequences of five additional species of the Triticeae tribe of grasses, wheat (*Triticum aestivum*) and its potential wild progenitors *Aegilops speltoides*, *Ae. tauschii*, and *T. urartu* plus rye (*Secale cereale*), also revealed the presence of *HvARM1*-like genes, suggesting a monophyletic origin of the partial gene-duplication event in a common Triticeae ancestor dating back at least 15 million (M) years [43] (Fig. 1e). To address the question of whether different evolutionary constraints act on *PUB15* and *ARM1* genes, we searched both orthologous gene groups for footprints of purifying or diversifying selection by calculating *d*_N_/*d*_S_ ratios (ω) at the codon level in a phylogenetic context. Protein sequence conservation among the seven species was high in both the U-box-containing N-terminal and the ARM-repeat-containing C-terminal parts of PUB15 (Additional file 1: Figure S3b), the existing polymorphisms being in agreement with phylogenetic species distances. By contrast, sequence conservation was reduced among ARM1 proteins (Additional file 1: Figure S3c), most clearly evident when comparing the two wild wheat species. As shown in Fig. 1f and Additional file 1: Figure S3d, both genes are subjected to purifying selection at the very N-terminus of ARM1 and within the ARM-repeat region. Selection was neutral in ARM1 outside these regions, whereas PUB15 sequences remained under purifying selection along the entire ARM1-overlapping part of the gene. This suggests that the function of ARM1 proteins is restricted to the binding of one or a few protein ligand(s) via their ARM repeats, whereas structural constraints on full-length E3 ligases that have to bind to substrate proteins and mediate the interaction with the highly conserved UBC domain of E2s are probably more stringent.

**Table 1:** Sequence overview of *HvPUB15* and its partial duplicate *HvARM1* in the barley genome

Identifier	*HvPUB15*	*HvARM1*
cDNA clone ID	HO23D08	HO14H18
HarvEST assembly #35 unigene Nr.	3072	3071
Full-coding sequence cDNA Acc. Nr.	AK361754	AK371875
Morex WGS contig Acc. Nr.^a^	CAJW010005672	CAJX010121345
Barke WGS contig Acc. Nr.^a^	CAJV010187631	CAJV010188692
Bowman WGS contig Acc. Nr.^a^	CAJX010851782	CAJX010121345
High-confidence barley gene ID^a^	HORVU3Hr1G113910	HORVU3Hr1G081380
Chromosome^b^	3HL	3HL
Position (Mbp)^b^	689.57	594.73
Syntenic to *Brachypodium distachyon*, *Oryza sativa*, *Sorghum bicolor*	No	No
Syntenic to *Ae. tauschii* ^c^	Yes	No

^a^Most significant BlastN result with 99–100% identity to genomic sequence of barley (http://webblast.ipk-gatersleben.de/barley/)

^b^Based on high-confidence (HC)-gene mapping of the barley reference sequence [72]

^c^Based on the *Ae. tauschii* genome 10.1073/pnas.1219082110

### Allelic variants of *HvARM1*

The phylogenetic and functional (see below) data of *ARM1* suggest that the gene is under selection for maintaining a quantitative level of resistance among Triticeae species to powdery mildew infection. We therefore analyzed gene variants (alleles) in a diverse collection of barley genotypes (Additional file 2: Tables S1–S3) for significant association with the severity of powdery mildew infection. Table 2 shows significantly associated single-nucleotide polymorphisms (SNPs) as well as gene-haplotype polymorphisms in two diverse, worldwide collections of barley landraces and cultivars. No association of *HvPUB15* gene variants with the same trait was found in these populations. This result supports the view that *HvARM1* — despite its partial nature — represents a functional gene protecting barley from powdery mildew attack, whereas the cellular functions of *HvPUB15* may be more complex.

### Function of HvARM1 during powdery mildew attack

**Fig. 2:**
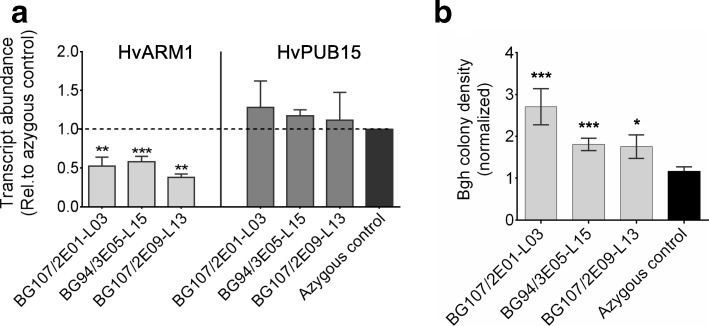
Silencing of *HvARM1* in *H. vulgare* affects quantitative resistance against *B. graminis* f.sp. *hordei*. **a** Transcript abundance of the *HvARM1* target gene and its possible off-target *HvPUB15* was determined by RT-qPCR in RNA from leaves of non-inoculated plants. Normalized transcript abundance relative to the *HvUBC* reference gene encoding an E2 ubiquitin conjugating enzyme (see Additional file 3: Methods S1) was further normalized to the mean value of azygous segregants (set to “1”). Mean values ± SE from 3 biological replicates (batches of plants sown on different dates) are shown. **b** Detached second leaves of T3 transgenic barley RNAi plants were inoculated with *Bgh* and infection was assessed microscopically 48 h after inoculation*.* Data represent normalized colony density (number/cm^2^/median of azygous control per experiment) ± SE from 2 to 3 biological replications. **a**, **b** Statistical differences between transgenic events and azygous plants are indicated by asterisks. *p < 0.05, **p < 0.005, ***p < 0.0005 (Student’s *t* test; two-tailed)

To validate the transient-induced gene silencing (TIGS) effect of *HvARM1* in the nonhost interaction with *Bgt* [39] and to further assess its role in the interaction with the adapted *Bgh*, we generated transgenic plants with silenced *HvARM1*. In rice, a detrimental effect of the knock-out mutation of *OsPUB15* was described that included severe growth retardation and seedling lethality [44]. A similar phenotype was observed in approximately 25% of transgenic barley events at the T1 generation (Additional file 1: Figure S4a). These individuals died after a few weeks, suggesting lethality caused by the homozygous transgene that may result in stronger silencing, in line with the failure to identify homozygous T2 or T3 lines. Homozygous lethality could reflect off-target silencing of the potentially housekeeping *HvPUB15* gene or an additional, more basal role of *HvARM1* in barley vegetative growth and development. The possible off-targeting of *HvPUB15* by the RNAi construct was addressed by software-based prediction and by off-target transcript quantification (Additional file 1: Figure S4b and Fig. 2a). Using default settings including end-stability difference and target-site accessibility thresholds, and the HarvEST:Barley assembly #35 sequence dataset [45], the software si-Fi21 (10.5447/ipk/2017/9) predicted 33 and 6 efficient 21-nt small interfering RNAs (siRNAs) for *HvARM1* and *HvPUB15,* respectively. However, the T3 transgenic lines consistently exhibited silencing of *HvARM1*, whereas no reduction of *HvPUB15* mRNA levels was found, suggesting that the transgene expression levels of the selected lines were not sufficient to silence the off-target.

Because *HvARM1* was discovered in a TIGS screen for attenuated NHR [39], we first tested T3 progeny of three selected events for susceptibility to *Bgt* (Additional file 1: Figure S4c). Although there was a considerable variability between individuals per line, two lines exhibited higher susceptibility to the non-adapted fungus as compared to the control group of azygous segregant plants. In general, azygous plants are considered as better controls since they have undergone the same transformation procedures and lost the transgenic construct by segregation. Indeed, the transformation procedure had an impact on the *Bgt* interaction because the azygous control group was on average more susceptible than the Golden Promise wild type. Figure 2b additionally shows that the three selected events were also more susceptible to *Bgh*, compared to a population of control plants consisting of azygous segregants plus progeny from three azygous individuals identified in the T2 generation.

Bombardment with gene-specific RNAi and with over-expression (OEX) constructs for a direct comparison of altered *HvARM1* versus *HvPUB15* expression levels revealed that gene-specific *HvARM1* silencing increased the relative susceptibility index (SI) to *Bgh* (Table 3), in line with the enhanced susceptibility observed in stable transgenic barley T3 plants. On the other hand, we found no significant effects of altering *HvPUB15* mRNA levels on the interaction of transformed cells with *Bgh,* again indicating more complex, homoeostatic rather than defense-related functions of the encoded protein. Following powdery mildew inoculation, endogenous transcript levels of *HvARM1* in peeled leaf epidermis were more strongly up-regulated above a basal level of expression compared to *HvPUB15* (Additional file 1: Figure S5), which also suggests a defense-related role of *HvARM1*.

### Localization and protein interactions of HvPUB15 and HvARM1

**Fig. 3:**
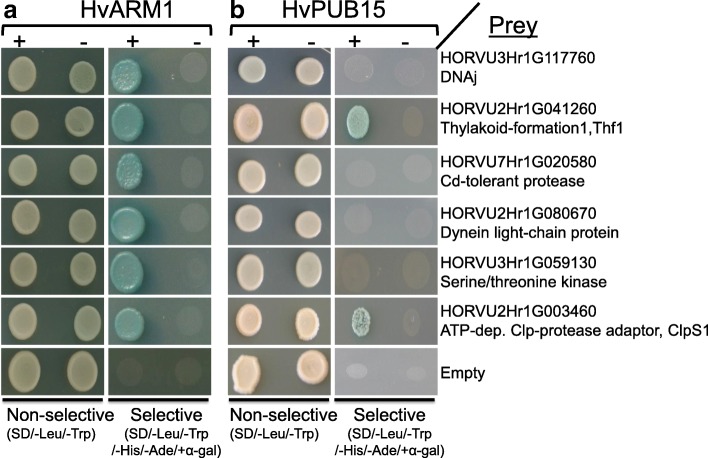
Proteins of *H. vulgare* interacting with HvARM1 and HvPUB15 in the yeast two-hybrid system (Y2H). **a** Full-length HvARM1 was used as bait in a Y2H screening of a cDNA library derived from *Bgh*-attacked barley leaves. Growth of yeast on SD-Leu/-Trp confirms the presence of both bait and prey vectors for protein expression. Growth on SD-Leu/-Trp/-His/-Ade indicates protein-protein interaction. No growth of empty bait vector + candidate prey confirms the absence of autoactivation of any prey construct for six final candidates. **b** Two of the six candidate protein interactors of HvARM1 also interact with full-length HvPUB15

Fusion proteins of HvARM1 and HvPUB15 with yellow fluorescent protein (YFP) showed a similar fluorescence pattern as non-fused YFP, suggesting nucleo-cytoplasmic localization (Additional file 1: Figure S6, panels a–c), in agreement with the localization of the proposed rice ortholog OsPUB15 [44]. Because the presence of the conserved ARM protein-protein interaction domain in HvARM1 suggests binding to other barley protein(s), we carried out a yeast two-hybrid screening in a prey library from *Bgh*-attacked barley leaves using HvARM1 as bait. This led to the identification of six barley proteins that interacted strongly and reproducibly with HvARM1 (Fig. 3a and Additional file 1: Table S8). Out of these six candidates the homologs of Clp-protease adaptor protein ClpS1 and Thylakoid formation 1 protein THF1 of *Arabidopsis thaliana* [46, 47] also strongly interacted with HvPUB15 (Fig. 3b) and may therefore be ubiquitination substrates of the E3 ligase. By using an in vitro ubiquitination assay we could show that HvPUB15 has ubiquitin ligase activity (Additional file 1: Figure S7). Because HvPUB15 catalyzed the polymerization of ubiquitin chains rather than auto-ubiquitination, it might be an E4 rather than an E3 ligase [48]. The possibility that HvThf1 and HvClpS1 are ubiquitination substrates for HvPUB15 was tested by transient OEX of the *HvPUB15* gene together with either *HvThf1:YFP* or *HvClpS1:YFP*, followed by the quantification of YFP-fluorescing cells 24 h after the bombardment. As shown in Table 4, co-expression with *HvPUB15* significantly reduced the number of HvThf1:YFP-fluorescing cells, suggesting that HvThf1 is an in vivo substrate to HvPUB15. By contrast, no indication of HvPUB15-mediated degradation of HvClpS1 was found. The possible involvement of these two HvARM1-interacting proteins in the interaction with *Bgh* was tested by TIGS and transient OEX. We observed a trend for reduced susceptibility by silencing and significantly enhanced susceptibility by OEX of *HvThf1* (Table 3), which may indicate that the proposed HvPUB15 substrate protein functions as a host susceptibility factor. Transient OEX of *HvClpS1* also enhanced susceptibility to *Bgh*, although there was no indication of the opposite effect by *HvClpS1* silencing. Co-localization experiments of HvThf1-YFP and HvClpS1-YFP C-terminal fusion proteins with the plastid marker Rubisco small subunit [49] confirmed their expected plastid localization (Additional file 1: Figure S6, panels d–k).

**Fig. 4:**
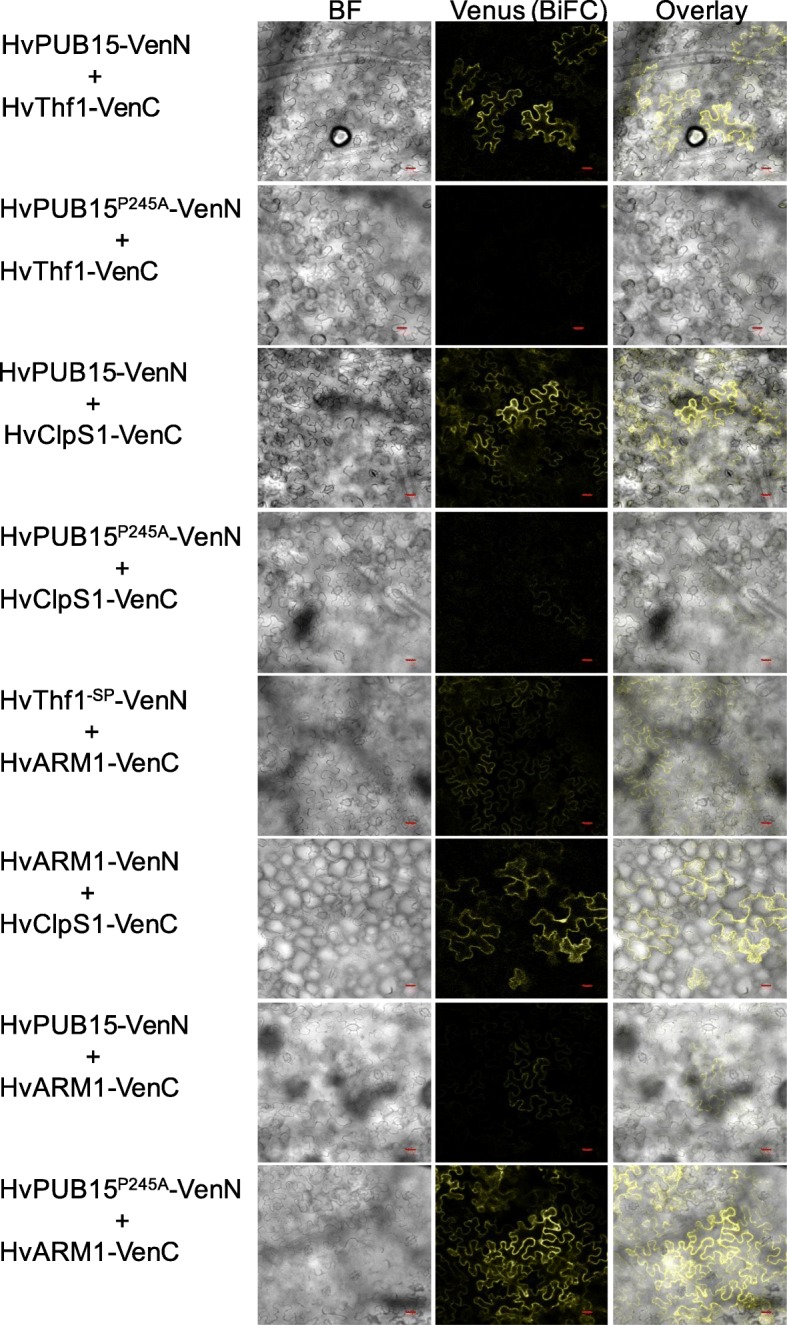
Bimolecular functional complementation (BiFC) of YFP by *H. vulgare* proteins interacting with HvARM1 and HvPUB15 in vivo. BiFC by HvPUB15- and HvARM1-interacting proteins in *Nicotiana benthamiana* leaves after infiltration of *A. tumefaciens* strains carrying the protein interaction partners fused C-terminally to split halves of YFP. *BF* bright field, *-SP* with deleted N-terminal plastid import signal, *VenN* N-terminal half of the stabilized YFP version “Venus”, *VenC* C-terminal half of the YFP “Venus”. Scale bars 20 μm

**Fig. 5:**
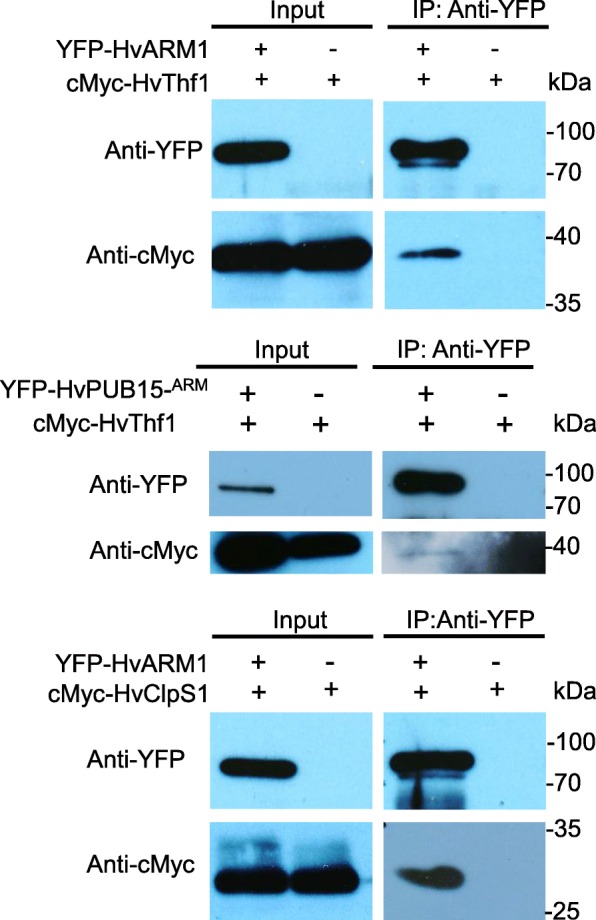
Co-immunoprecipitation (Co-IP) of *H. vulgare* proteins interacting with HvARM1 and HvPUB15 in vivo. Co-IP of antibody-tagged barley proteins in *A. thaliana* mesophyll protoplasts. YFP-fused *HvARM1* (77.5 kD), and *HvPUB15* (83.2 kD) were co-expressed with cMyc-tagged HvThf1 (40 kD) and *HvClpS1* (30 kD), respectively, for each interaction. Co-IP was performed using anti-YFP antibodies and total proteins extracted from *A. thaliana* protoplasts

In vivo interaction of HvARM1, HvPUB15, and HvPUB15^ARM^ (the ARM domain of HvPUB15) with HvThf1 and HvClpS1 was assessed by split-YFP bimolecular functional complementation (BiFC) assays in *Agrobacterium*-infiltrated *Nicotiana benthamiana* leaves and by co-immunoprecipitation in *A. thaliana* protoplasts. Figure 4 shows that the transient co-expression of HvPUB15 or HvPUB15^ARM^ with either HvClpS1 or HvThf1 gave rise to BiFC (YFP) signals primarily in epidermal cells. The localization patterns of the fluorescence signals indicated that the proteins interacted in the cytoplasm, which was confirmed by the absence of co-localization with the plastid marker protein 35S:SSU_1–79_–mCherry. The BiFC signals were abolished or strongly reduced by using the U-box mutant HvPUB15^P245A^ as an interaction partner, suggesting specificity of the interaction. HvARM1 also interacted with HvClpS1 and HvThf1. Moreover, interactions were observed between HvPUB15 and HvARM1, and here the HvPUB15 U-box mutation increased BiFC signals instead of reducing them. The specificity of the interaction was further confirmed using additional controls and quantitative fluorescence measurement (Additional file 1: Figures S8 and S9). Co-immunoprecipitation experiments in *A. thaliana* protoplasts of cMyc-tagged HvARM1 and HvPUB15^ARM^ together with either HvThf1 or HvClpS1 confirmed in vivo interaction of HvPUB15^ARM^ with HvThf1, HvARM1 with HvThf1, and HvARM1 with HvClpS1 (Fig. 5). Taken together, the results suggested in vivo interaction of HvPUB15 with HvClpS1 and HvThf1, whereby the presence of an intact U-box was required for the interaction. In addition, HvARM1 as well as the ARM -domain of HvPUB15 interacted with HvClpS1 and HvThf1. One of the HvPUB15-interacting proteins, HvThf1, was identified as a potential ubiquitination substrate and as a host susceptibility factor to *Bgh*.

### Genome-wide search for expressed partial gene duplicates

**Fig. 6:**
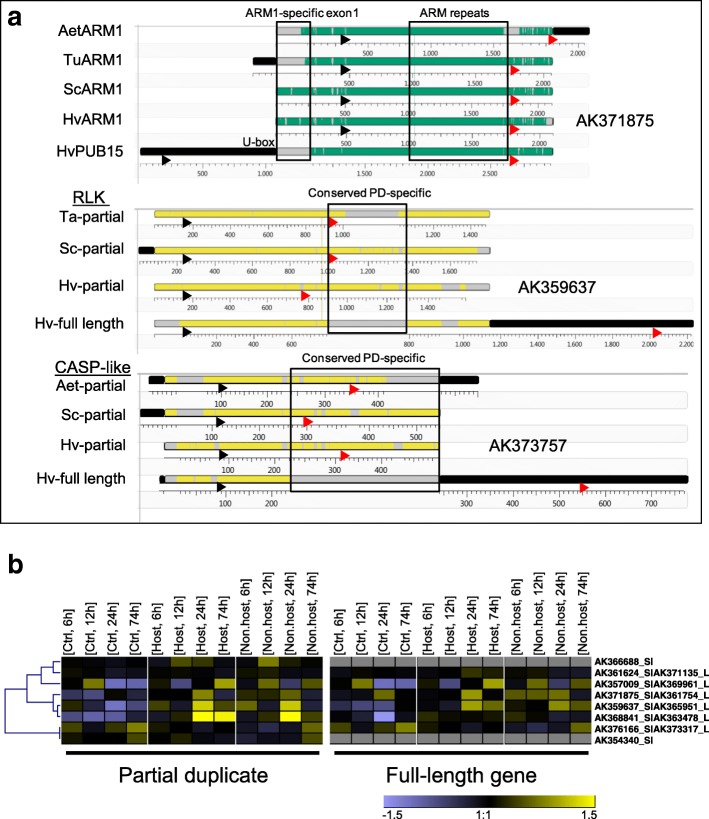
Genome-wide analysis of expressed and conserved gene duplicates. **a** Pairs of barley cDNA encoding full-length proteins and partial copies, respectively, which are conserved across Triticeae, were aligned using MAUVE algorithm. The *dark green shading* in ARM1 or *yellow shading* in the rest indicates aligning sequences, whereas *gray shading* shows gaps. *Black* and *red arrowheads* indicate start- and stop-codon positions of the corresponding open reading frames, respectively. Non-aligning sequences (*in black*) at the beginning or end were automatically detached from the alignment blocks. Only the first 500 bp of the CASP-like duplicates giving rise to truncated proteins are shown.  Scale below alignments, sequence length in bp; *PD* partial duplicate. *Aet*, *Aegilops tauschii*; *Tu*, *Triticum urartu*; *Sc*, *Secale cereale*; *Hv*, *Hordeum vulgare*. For annotation and further details see Additional file 2: Table S4. **b** Transcript regulation in peeled barley leaf epidermis by *Bgh* (adapted host pathogen) or *Bgt* (nonhost pathogen). Total RNA was isolated at the time indicated after inoculation and hybridized to the Barley Gene Expression Array of Agilent. For the link of cDNA accession number to Agilent probe IDs, see Additional file 2: Table S4. Transcript data have been submitted to ArrayExpress (Acc. E-MTAB-2916). Hierarchical clustering of gene-median-centered, normalized signal intensities is shown. The color scale ranges from log(2)-1.5 to 1.5. Mean signal intensities from three independent inoculation experiments are shown

Does the partially duplicated gene pair of *PUB15* and *ARM1* represent a unique case in Triticeae genome evolution, or could we find indications for additional partial gene duplicates with putative functions? To address this question we conducted a genome-wide search for pairs of full-length complementary DNA (cDNA) sequences with high sequence similarity but clearly different lengths of their longest open reading frames. The rationale behind this approach was to select for partially duplicated or rearranged genes that are expressed and therefore more likely to fulfill a biological role. Starting with a library of 23,614 full-length cDNA sequences (http://barleyflc.dna.affrc.go.jp/bexdb/), we found 1154 matching cDNA pairs with a sequence identity of 80–99%. A subsequent tBlastx analysis of these pairs revealed 205 pairs with a length difference of matching open reading frames of > 25%. After further filtering steps to exclude non-spliced transcripts and chimeric as well as partial clones, we identified eight expressed pairs of putative, partially duplicated genes including *HvPUB15/HvARM1* (Additional file 2: Tables S4 and S5 and Additional file 1: Figure S13). A majority of these are localized at non-tandem positions in the barley genome (five or more gene models apart from each other, or on different chromosomes). Three genes encode proteins from families known to be involved in plant-pathogen interactions: PUB15 plus two receptor-like kinases. Besides the pair of *ARM1* and *PUB15* we found two more gene pairs giving rise to full-length and truncated proteins, respectively, which are conserved across Triticeae species (Fig. 6a). The truncated open reading frames of one of these pairs encoding a receptor-like kinase are caused by a 19-bp deletion in barley reconstituting a stop codon, or by stop codon mutations approximately 200 bp further downstream in *T. aestivum* and *S. cereale*, which mark the insertion of a non-coding genomic sequence. The truncated open reading frames of the second pairs are caused by the insertion of approximately 2 kb of a non-coding sequence. Finally, although this was not a criterion for their selection, several transcripts encoding truncated proteins were up-regulated by powdery mildew attack (Fig. 6b and Additional file 2: Table S6). Therefore, these rearranged or partial gene duplicates in addition to *HvARM1* might have evolved to fulfill new functions in Triticeae species.

## Discussion

At least six species of the Triticeae tribe of grasses possess *ARM1,* a partial gene copy of a U-box/ARM-repeat E3 ligase closely related to *OsPUB15* of rice [44]. The rice genome also contains a number of “ARM-repeat only” genes, but none of them appears to represent a partial copy of *OsPUB15*, because BlastN analysis of the ARM-repeat region of *OsPUB15* (positions 2000–2952 in cDNA Acc. AK106557.1) at the National Center for Biotechnology Information (NCBI) did not produce significant hits for any other rice gene. By contrast, the same query sequence revealed *HvARM1* as the most significant hit (86% identity) in barley. Sequence analysis in cultivated barley, wheat, and rye, and in three diploid wild wheat species suggest a monophyletic origin of *ARM1.* The large and highly repetitive genomes of the cultivated Triticeae species barley, wheat, and rye are known to be rich in gene-like sequences including partial duplicates, and most of them were classified as putative pseudogenes [19, 20]. The classification criteria for these putative pseudogenes were (1) non-syntenic map positions among grasses and (2) unique occurrence in one species or in one of the three subgenomes of hexaploid wheat. Illegitimate meiotic crossing over and subsequent sequence capture by transposable elements, as well as random sequence insertion during non-homologous end joining for double-strand break DNA repair, are the two proposed major events leading to non-tandem (partial) gene duplicates [50]. By contrast, sub- or neo-functionalized, expressed and full-length gene duplicates often exist as tandemly repeated gene pairs or clusters of genes, as a result of unequal crossover during meiosis that is often followed by gene conversions [51, 52]. As shown in Table 1, the full-length genes *HvPUB15* and *AetPUB15* share syntenic map positions on the long arm of homologous chromosome group 3 [53, 54]. The partially duplicated *HvARM1* gene was mapped at a distance of approximately 95 Mbp from *HvPUB15* on chromosome 3H, and all six analyzed Triticeae *ARM1* genes contain a non-repetitive, unknown sequence in exon 1 that is not present in the corresponding *PUB15*-like genes. Taken together this suggests that an event of DNA double-strand break repair in a common ancestor of Triticeae species gave rise to *ARM1*.

Gene duplication followed by neo-functionalization is a source of generation of new genes in plants and has been reported for individual genes or at a genome level [55–58]. However, there is no report of a “partial” gene duplication followed by neo-functionalization, and our results presented here suggest that *ARM1* escaped pseudogenization and took over a new biological function in defense against powdery mildew fungi and perhaps other pathogens: First, the genomes of six species belonging to four different genera maintained the partial gene copy with a high degree of sequence conservation at the ARM-repeat region. Second, in all six species *ARM1* is supported by perfectly matching expressed sequence tag (EST) sequences or other transcriptome data, demonstrating that the corresponding genes are actively transcribed. In barley, transcript regulation data suggest a gain of function of *HvARM1* in terms of a more pronounced pathogen-induced accumulation in the epidermis compared to *HvPUB15* (Additional file 1: Figure S5). Third, all *ARM1* sequences are characterized by intact open reading frames starting approximately in the middle part of the PUB15 protein and extending to its C-terminus. Further, ARM1 is subjected to a purifying selection at the N-terminus and within the ARM-repeat region, while the selection is neutral outside these regions (Fig. 1f). This proposes that the function of ARM1 proteins is probably restricted to protein-protein interactions via their ARM repeats, where maintaining a high degree of sequence conservation seems to be of a certain evolutionary importance. Due to their non-functional nature and no selective pressure, the ratio of non-synonymous to synonymous amino acid substitution value should be ~ 1 for pseudogenes. In contrast, the ARM1 values are well below 1 in the overlapping region of PUB15 (Fig. 1f). Four, transient OEXs of *HvARM1* without the predicted ATG translation-initiation codon (*HvARM1*^*∆ATG*^) eliminated the transgene effect, demonstrating that it was caused by translated HvARM1 protein (Additional file 1: Figure S14). Mutating randomly selected but highly conserved amino acids in the ARM-repeat domain of HvARM1 (HvARM1^L286H^ and HvARM1^L308K^) significantly affected the relative SI towards susceptibility to *Bgh* (Additional file 1: Figure S14). This suggests that the ARM-repeat domain in HvARM1 is essential for its function. Fifth, allelic variants of *HvARM1* were found to be significantly associated with the severity of powdery mildew infection in collections of locally adapted barley landraces and diverse cultivars (Table 2 and Additional file 2: Table S1). In both collections the most significant SNPs were associated with clear and statistically significant differences in *Bgh* infection (41% versus 57%, *p* = 0.00037 in collection WHEALBI_LRC; 35% versus 51%, *p* = 0.023 in collection WHEALBI_CULT). The significant SNP in the landrace collection was located in the 5′ untranslated region of the HvARM1 transcript, whereas the significant SNP in the cultivar collection causes a glycine-to-valine change at position 437 of the encoded protein. The cultivars carrying the corresponding significant, resistance-associated haplotype H01 were derived from very different regions of the world and therefore probably not similar by descent. The complete absence of association of *HvPUB15* alleles with *Bgh* infection furthermore supports the view that the E3 ligase primarily has important housekeeping functions such as quality control and turning over of plastid-localized proteins [59], with no adaptation flexibility during pathogen co-evolution.

**Table 2:** Marker-trait associations of *HvARM1* and *HvPUB15* in diverse collections of cultivated *H. vulgare* ssp. *vulgare*

Gene	Population^a^	Trait^b^	Marker^c^	Minus log(*p*)^d^	Holm corr. *p*^e^
*HvARM1*	WHEALBI_LRC	PM_ max_2_isol rel_Rol	H02	2.893	**0.0051**
*HvARM1*	WHEALBI_LRC	PM_ max_2_isol rel_MRX	H02	2.848	**0.0057**
*HvARM1*	WHEALBI_LRC	PM_JKI_75_rel_MRX	H02	2.418	**0.0153**
*HvARM1*	WHEALBI_LRC	PM_ max_2_isol rel_Rol	S3H_594732776	2.893	**0.0051**
*HvARM1*	WHEALBI_LRC	PM_ max_2_isol rel_MRX	S3H_594732776	2.848	**0.0057**
*HvARM1*	WHEALBI_LRC	PM_JKI_75_rel_MRX	S3H_594732776	2.418	**0.0153**
*HvARM1*	WHEALBI_CULT	PM_JKI_75_rel_Rol	S3H_594731277	3.826	**0.0006**
*HvARM1*	WHEALBI_CULT	PM_JKI_75_rel_MRX	S3H_594731277	3.460	**0.0014**
*HvARM1*	WHEALBI_CULT	PM_ max_2_isol rel_MRX	S3H_594731277	3.374	**0.0017**
*HvARM1*	WHEALBI_CULT	PM_JKI_75_rel_Rol	H01	3.826	**0.0004**
*HvARM1*	WHEALBI_CULT	PM_JKI_75_rel_MRX	H01	3.460	**0.0010**
*HvARM1*	WHEALBI_CULT	PM_ max_2_isol rel_MRX	H01	3.374	**0.0013**
*HvPUB15*	WHEALBI_LRC	PM_JKI_242_rel_Rol	H10	0.871	0.5379
*HvPUB15*	WHEALBI_LRC	PM_JKI_242_rel_Rol	H11	0.684	0.6203
*HvPUB15*	WHEALBI_LRC	PM_ max_2_isol rel_MRX	H10	0.599	1
*HvPUB15*	WHEALBI_LRC	PM_JKI_242_rel_Rol	S3H_689574119	0.814	1
*HvPUB15*	WHEALBI_LRC	PM_JKI_242_rel_Rol	S3H_689574678	0.814	1
*HvPUB15*	WHEALBI_LRC	PM_JKI_242_rel_Rol	S3H_689575062	0.814	1
*HvPUB15*	WHEALBI_CULT	PM_ max_2_isol rel_Rol	S3H_689573944	1.055	1
*HvPUB15*	WHEALBI_CULT	PM_JKI_75_rel_Rol	S3H_689573944	1.002	1
*HvPUB15*	WHEALBI_CULT	PM_JKI_75_rel_MRX	S3H_689573776	0.925	1
*HvPUB15*	WHEALBI_CULT	PM_JKI_75_rel_MRX	H10	0.511	0.926
*HvPUB15*	WHEALBI_CULT	PM_ max_2_isol rel_MRX	H10	0.372	1
*HvPUB15*	WHEALBI_CULT	PM_ max_2_isol rel_Rol	H11	0.359	1

Significantly associated SNP- as well as gene-haplotype polymorphisms are indicated in bold

^a^*CULT* cultivars, *LRC* landraces

^b^Three different powdery mildew (PM) traits were recorded: (1) infection caused by isolate JKI_75 relative to internal reference genotypes Morex (MRX) or Roland (Rol), (2) infection caused by isolate JKI_242 relative to MRX or Rol, (3) maximum infection caused by either isolate relative to MRX or Rol

^c^Per population and gene the three most significant haplotype-trait as well as SNP-trait associations are shown; *H* haplotype, *S* SNP

^d^Negative log(10) of *p* value for the null hypothesis of a marker-trait association

^e^Values > 1 of multiple-testing corrected *p* values are replaced by 1; number of SNPs or haplotypes per gene = number of tests.

Functional tests of *HvARM1* by transient OEX in wheat suggested a resistance-related role during the interaction with adapted and non-adapted powdery mildew fungi (Table 5). Similar to the results presented here, a resistance-enhancing effect was found by OEX of the ARM domain of the *AtPUB13* gene in *A. thaliana,* which is involved in protein degradation of the flagellin PAMP receptor FLS2 [60]. Transgenic plants ectopically over-expressing only the ARM domain of the *AtPUB13* phenocopied the *atpub12/13* double-mutant effect of enhanced pathogen resistance by blocking the AtPUB12/13-mediated FLS2 degradation. In the current study, our data point out that in barley and other Triticeae species, a novel partial gene-duplication event followed by neo-functionalization of *ARM1* has resulted in expression of a single ARM domain as a natural mechanism for enhancing disease resistance to powdery mildew. Further, a study in human antiviral immunity response suggests that partial duplication of protein-protein interaction domains could facilitate the extension of novel immune signaling pathways [18]. Besides a putative antagonistic role to PUB15 (see below), ARM1 might also function as a decoy inactivating or trapping pathogen effectors [61]. The recent report of partial effector target-gene duplicates fused to NB-LRR-type resistance genes, which function as single-domain decoys, is reminiscent of the single-domain *ARM1* gene described here, although the latter exists as an unfused partial copy [62]. Regarding the full-length gene *PUB15*, a defense-related role was proposed in rice because OEX of *OsPUB15* caused spontaneous defense responses and increased pathogen resistance [63]. We could not confirm a defense-related role of *HvPUB15* by TIGS or transient OEX in *Bgh*-attacked barley (Table 3), but this might reflect multiple and balancing effects of its mis-expression, especially if it fulfills additional, housekeeping functions. The reported lethality of *OsPUB15* mutations in rice [44] supports *PUB15* as a housekeeping gene, which might be co-opted by *Bgh* for host accommodation.

**Table 3:** Effect of TIGS and transient over-expression (OEX) of *HvPUB15*, *HvARM1*, and genes encoding their interacting proteins on QR against *B. graminis* f.sp. *hordei*

Bombarded gene	Proposed function	TIGS			Transient OEX		
		Rel. SI (log2)^a^	*p* (*t* test)^b^	*n* ^c^	Rel. SI (log2)^d^	*p* (*t* test)^b^	*n* ^c^
*HORVU3Hr1G113910*	U-box/ARM E3 protein ligase (HvPUB15)	0.17 ± 0.41	0.6950	5	0.26 ± 0.19	0.2316	7
*HORVU3Hr1G081380*	ARM-repeat protein (HvARM1)	**1.16 ± 0.22**	**0.0126**	**4**	−0.01 ± 0.21	0.9516	5
*HORVU2Hr1G041260*	Thylakoid formation 1 (Thf1)	−1.35 ± 0.59	0.0689	6	**0.47 ± 12.1**	**0.0035**	**6**
*HORVU2Hr1G003460*	ATP-dependent Clp-protease adaptor (ClpS1)	−0.60 ± 0.41	0.2168	5	**0.70 ± 0.06**	**8.43E-5**	**6**
*HORVU7Hr1G008760* ^e^	Syntaxin HvSNAP34	**1.14 ± 0.25**	**0.0050**	**5**			
*TaPrx103* ^f^	Class III peroxidase TaPrx103				**−1.06 ± 0.15**	**6.61E-6**	**14**

^a^Relative to the pIPKTA30 internal empty vector control

^b^One-sample *t* test (two-tailed) of log2-transformed relative susceptibility index (SI) against the hypothetical value “0”.

^c^Number of independent bombardment experiments

^d^Relative to the pIPKTA09 internal empty vector control

^e^TIGS of this target gene enhances susceptibility to *Bgh* and served as positive control [93]

^f^Over-expression of this gene enhances resistance in barley and wheat against *B. graminis* and served as positive control [106]

Statistically significant effects are highlighted in bold

**Table 4:** Degradation of putative HvPUB15 substrate proteins by transient OEX of HvPUB15

Co-bombarded plasmids	Thf1:YFP			ClpS1:YFP		
	Rel. cell no. (log2)^a^	*p* (*t* test)^b^	*n* ^c^	Rel. cell no. (log2)^a^	*p* (*t* test)^b^	*n* ^c^
pIPKTA09	0	–	3	0	–	4
HvPUB15	**−0.59 ± 0.11**	**0.0365**	**3**	0.03 ± 0.28	0.9030	4
HvPUB15 + HvARM1	**−0.42 ± 0.07**	**0.0327**	**3**	−0.64 ± 0.56	0.3334	4
HvARM1	−0.30 ± 0.31	0.4357	3	0.33 ± 0.19	0.8815	3

^a^Number of YFP-fluorescing cells (relative to the empty vector control pIPKTA09) expressing HvThf1:YFP or HvClpS1:YFP constructs in the presence of different co-bombarded plasmids

^b^One-sample *t* test (two-tailed) against the hypothetical value “0”

^c^Number of independent bombardment experiments

Statistically significant effects are highlighted in bold

**Table 5:** Transient over-expression of *HvARM1* enhances resistance in *T. aestivum* against *B. graminis* f.sp. *tritici*

Bombarded gene	Relative SI (log2)^a^	*p* (*t* test)^b^	*n* ^c^
*HvARM1*	−0.37 ± 0.12	0.0107	16
^d^ *HvARM1* ^*∆ATG*^	0.19 ± 0.13	0.2168	5

^a^Susceptibility index, normalized to the empty vector control pIPKTA09 and log2-transformed

^b^One-sample *t* test (two-tailed) of log2-transformed relative SI against the hypothetical value “0”

^c^Number of independent bombardment experiments

^d^Negative control construct without translation start codon

The HvARM1 and HvPUB15 proteins interacted in yeast and in plants with the plastid-localized proteins HvClpS1 and HvThf1, and both appear to be susceptibility-related factors based on TIGS and transient OEX results (Table 3). The observation that transcripts of both *HvClpS1* and *HvThf1* were down-regulated in the epidermis of powdery mildew-attacked leaves might reflect an attempt of the plant to reduce the levels of susceptibility-related factors (Additional file 1: Figure S10 and Additional file 2: Table S7). In contrast to HvClpS1, the degradation of HvThf1 appeared to be mediated by HvPUB15, which suggests this protein as the strongest candidate for a susceptibility-related process involving HvPUB15 and being antagonized by HvARM1. However, because *HvPUB15/HvARM1* co-expression did not suppress PUB15-induced HvThf1:YFP degradation, we cannot propose a simple model for a direct antagonistic mode of action of HvARM1. The THF1 protein of *A. thaliana* was found to be localized in the plastid stroma and at its outer membrane facing the cytoplasm, where it was proposed to play a role in sugar sensing [46]. This is relevant with respect to the high demand of powdery mildew-infected cells for energy equivalents to transport large amounts of glucose into haustoria, a process that depends on SWEET sugar transporters and other factors [64–66]. Further support for the involvement of *Thf1* in disease responses comes from the finding that the closest wheat homolog to *HvThf1*, designated as *TaToxABP1,* is a binding protein and a target of Toxin A produced by the necrotrophic, tan-spot fungal pathogen *Pyrenophora tritici-repentis* [67]. Toxin A treatment also triggered an oxidative burst in leaves of wheat and barley [68, 69], thereby providing a link of *Thf1* function with reactive oxygen species (ROS) control, at least in chloroplasts, and proposes a mode of action of Toxin A. Also, the interaction of the Thf1 protein with I2-like coiled-coil (CC) domains of several NB-LRR-type resistance proteins leading to their destabilization has been reported [70]. Finally, the link of protein turnover by proteasomal degradation with chloroplast biology was recently established by reports on the role of the closest *HvPUB15* homolog in *A. thaliana* designated as *AtPUB4*, and of *AtCHIP,* in plastid quality control and degradation of the caseinolytic plastid peptidase AtClpP4, respectively [42, 59, 71]. Mutants of *AtPUB4* showed reduced resilience against abiotic stress, indicative of compromised plastid-based control of ROS generation. Plants silenced in or over-expressing *AtCHIP* exhibited a chlorotic phenotype indicating a strict requirement of accurate control of AtClpP4 levels for cellular homoestasis. In contrast to Thf1, no published information supporting a role of ClpS1 in plant-pathogen interactions is currently available.

Besides the *PUB15/ARM1* gene pair, we found evidence for seven additional gene-duplication events across the barley genome that gave rise to novel, expressed genes encoding truncated proteins (Additional file 1: Figure S13). This number may be underestimated because the search was based on a library of 23,614 full-length cDNA clones, which covers approximately 50–66% of the entire predicted gene space. A more comprehensive study of partial gene duplications will have to await improved gene models of the barley reference sequence, as compared to the current annotation [72]. The *PUB15/ARM1* gene pair may also not be the only case of evolutionarily conserved duplication/gene rearrangements in Triticeae, because we also found similarly conserved events in a receptor-like kinase and a CASP-like protein (Fig. 6). Future expression studies and functional tests of these partially duplicated or rearranged genes across Triticeae may reveal a more comprehensive picture of their potential to support host survival.

## Conclusion

The results presented here suggest that *ARM1* is a case of gene neo-functionalization after a non-tandem, partial gene-duplication event that gained a role in quantitative resistance against *B. graminis* and maybe other pathogenic fungi. The *ARM1* most likely originates from a partial duplication of the E3 ligase *PUB15,* which occurred in a common ancestor of the *Triticeae* tribe of grasses. At least in barley, the HvARM1-interacting protein and proposed substrate of HvPUB15, the plastid-localized HvThf1, links susceptibility to biotrophic pathogens with homeostatic protein function in plastids. Our findings shed new light on a novel mechanism to employ partial duplication of the protein-protein interaction domain to facilitate the expansion of immune signaling networks. The genome-wide search for further neo-functionalized gene duplicates encoding truncated proteins may uncover a yet poorly explored aspect of plant genome dynamics, which might be relevant for plant-stress responses in general and for plant-pathogen co-evolution in particular.

## Methods

A more detailed description of materials and methods used in this study is provided in Additional file 3: Methods S1.

### Plant and fungal material

TIGS and transient OEX experiments were done in 7-day-old seedlings of spring barley Golden Promise, except for OEX of site-directed mutagenesis (SDM) and TIGS of HvThf1 and HvClpS1 where the closely related genotype Maythorpe was used. Stable transgenic barley plants of cv. Golden Promise were generated as described [73]. Bombarded leaf segments or transgenic plants were inoculated with Swiss *Bgt* field isolate FAL 92315, or Swiss *Bgh* field isolate CH4.8 throughout the study.

### Sequence alignment, phylogenetic analysis, and estimation of pressures of selection

*PUB15* and *ARM1* orthologs were obtained from five Triticeae species, besides barley, including *Secale cereale* [21, 74], *Triticum aestivum* [75], and its three potential wild progenitors *T. urartu* (A genome), *Aegilops speltoides* (B genome), and *Ae. tauschii* (D genome). The sequences were retrieved by BlastN search of the *H. vulgare* sequences (AK361754 and AK371875, respectively) against the whole genome assembly databases (http://webblast.ipk-gatersleben.de/ryeselect/ for *S. cereale* and https://urgi.versailles.inra.fr/blast/blast.php for *T. aestivum* and its relatives). The rice ortholog, *OsPUB15* (XM_015795011), was downloaded as outgroup sequence. All 17 coding sequences could be fully acquired but for the *Ae. speltoides ARM1* sequence lacking the last exon.

The sequences were aligned with MAFFT v7.308 [76] using the default settings within Geneious 10.0.9 (https://www.geneious.com) [77] followed by manual adjustment. The model of sequence evolution was determined with jModeltest 2.1.10 [78]. The best-fit model, identified with the Akaike information criterion AIC [79, 80], was the general time-reversible (GTR) [81] with rates variation according to a gamma distribution [82]. The maximum likelihood (ML) phylogenetic tree was calculated with RAxML v8.2.7 [83] using the GTRGAMMA model and 100 bootstrap replicates (options –f a and –x).

Pressures of selection were investigated at the codon level throughout the phylogeny for both proteins separately. The pressure of selection can be estimated by ω (non-synonymous substitution rate divided by synonymous substitution rate, *d*_N_/*d*_S_). An ω < 1 suggests that the site is under negative, or purifying, selection, while an ω > 1 indicates that positive selection is occurring, and ω = 1 for neutral changes. Values of ω were estimated for each non-ambiguous codon using the codeml program within the package PAML4.9 h [84]. Following Jeffares et al. [85], codon site models M0 [86, 87], allowing only one class of ω, M1a [88, 89], allowing two categories of sites (0 < ω_0_ < 1 and ω_1_ = 1 with proportions p_0_ and p_1_ = 1- p_0_), and M2a [88, 89], which includes a proportion of sites under positive selection, were tested. Likelihood ratio tests (LRTs) were performed to compare models M0 and M1a, as a test for variation of ω among sites, and models M1a and M2a, as a test for positive selection, against a χ^2^ distribution (with one and two degrees of freedom, respectively). Both proteins showed variations of ω among sites with M1a versus M0 LRTs significant. But with nearly identical likelihoods for models M1a and M2a, *PUB15* and *ARM1*, though to a lesser extent, proved to be under purifying selection with a small portion of sites under neutral selection. Sequence information is available at 10.6084/m9.figshare.c.4092686.v1.

### Exome capture sequencing

Genomic DNA was extracted from barley leaf material from a single plant for each accession and used for the hybridization with the barley SeqCap Ez oligo pool (Design Name: 120426_Barley_BEC_D04, [54]. Quality-trimmed reads were mapped to the reference genome (http://webblast.ipk-gatersleben.de/barley_ibsc/downloads/) with Burrows-Wheeler Aligner (BWA) v0.7.5a using the mem algorithm with default parameters [90] and retaining only properly paired reads. Variant calling and realignment around indels were performed with Genome Analysis Toolkit (GATK), version 2.7.4 (https://software.broadinstitute.org/gatk/). Variant calls were filtered for high quality and ≥ 80% of samples being represented at each locus, and a dataset of 449,585 SNPs was produced, suitable for genetic association analysis of the two genes under investigation (full information about genome-wide variants from this dataset will be published elsewhere).

### Association genetic analysis

Association of SNP and gene haplotypes (marker) of *HvARM1* and *HvPUB15* with the severity of *Bgh* infection (trait) was calculated based on genetic and phenotypic data of two diverse collections of cultivated barley (*H. vulgare* ssp. *vulgare*). *Bgh* infection values were determined in a detached leaf assay using second leaves of approximately 12-day-old seedlings, as described [91]. First, a worldwide collection of 76 landraces (WHEALBI_LRC) was inoculated either with isolate JKI-75 or JKI-242, which exhibit a complex and complementing virulence spectra [92]. Second, a worldwide collection of 127 cultivars (WHEALBI_CULT) was inoculated with the same two *Bgh* isolates. Both populations consisted of single seed-derived lines, and an average of 5 parallel plants per line was used in each inoculation assay. For passport data of all lines see Additional file 2: Table S1. Seven days after inoculation, disease was scored by estimating the percentage of leaf area covered by fungal mycelium. Because disease scores were variable between different inoculation experiments, they were normalized to internal standards cv. Roland or Morex, as indicated. Phenotypic data of all isolate-genotype combinations are based on two independent inoculation series. SNP calls were derived from exome capture resequencing, and haplotypes were calculated based on the combination of SNP calls per gene. SNP-trait and haplotype-trait associations were calculated in TASSEL v4.1 using a mixed linear model with kinship as random effect. Marker data for kinship calculations were derived from 4032 polymorphic genotyping by sequencing (GBS) markers. Marker-trait associations were assumed significant if the Holm’s-corrected *p* value was < 0.05 (number or SNP or haplotypes/gene = number of tests).

### TIGS and transient over-expression

TIGS constructs were generated and transferred by particle bombardment into leaf epidermal cells of 7-day-old barley seedlings as described [93]. Leaf segments were inoculated 3 days after the bombardment with *Bgh* at a density of 140–180 conidia mm^–2^. Transformed GUS-stained epidermal cells as well as haustoria-containing transformed (susceptible) cells were counted 48 h after inoculation, and TIGS effects on the susceptibility index (SI) were statistically analyzed [91].

For verification of transgene effects, *HvARM1* was excised from a subclone of the bacterial artificial chromosome (BAC) HVVMRXALLhA0581d24 (Acc. Nr. KM979563) as a *Stu*I/*Sph*I fragment and inserted into *Sma*I/*Sph*I sites of pIPKTA09 [94]. For transient OEX of candidate genes*,* full-coding sequences were polymerase chain reaction (PCR) amplified from cDNA and inserted as an *Xba*I fragment into the multiple cloning site of pIPKTA09. Mutations (HvARM1^-L286H^ and HvARM1^-L308K^) were introduced by SDM using the QuikChange Kit (Stratagene, San Diego, CA, USA). The resulting sequence-verified constructs were bombarded into barley as described for BAC clones. For PCR primers used in this study see Additional file 1: Table S9.

For the HvThf1 and HvClpS1 protein degradation assay, 4 μg each of plasmid DNA encoding HvPUB15 or HvARM1 plus Thf1:YFP or ClpS1:YFP plus pUbiGUS [93] were co-bombarded into 7-day-old barley cv. Golden Promise. The numbers of YFP-fluorescing cells with plastid-localized signals were counted 24 h after particle bombardment, followed by GUS-staining [93]. The numbers of GUS-expressing cells were used for normalization of the YFP signal.

### Inoculation and evaluation of transgenic plants

Phenotypic evaluation of *Bgh* and *Bgt* interactions was done microscopically on second, detached leaves of 12–14 day-old plants placed on phytoagar plates (23,2 cm × 23,2 cm) inoculated at a spore density of 30–40 conidia mm^− 2^. Inoculated leaf segments were incubated for 48 h (*Bgh*) or 72 h (*Bgt*) followed by staining with Coomassie brilliant blue R 250 [95]. The number of growing colonies/leaf area was counted under a standard bright field microscope at 100× magnification.

### Yeast two-hybrid experiments

Yeast two-hybrid screening was performed according to the Yeast Handbook and manual of Matchmaker™ Library Construction and Screening Kits (Takara/Clontech Laboratories, Saint-Germain-en-Laye, France). The full-length coding sequence of HvARM1 (1–442 AA) was used to screen a library of 7 × 10^6^ mating events according to [96]. For targeted Y2H assays, the coding region (1–831 AA) of HvPUB15 was used to test positive prey clones of the HvARM1 screening.

### Bimolecular fluorescence complementation and co-immunoprecipitation

For bimolecular fluorescence complementation (BiFC) of HvARM1 and HvPUB15 proteins with potential plastid interactors, *Nicotiana benthamiana* plants were grown and agro-infiltrated as described in detail in Additional file 3: Methods S1. For BiFC with *HvThf1* and *HvClpS1*, the wild-type full-length sequences of *HvPUB15* or *HvARM1*, U-box mutants of *HvPUB15*, the ARM domain (351 to 831 AA) only of *HvPUB15*, or *HvThf1* without N-terminal plastid import signal (-SP) were cloned into 35S::^GW^VYNE-pBar and 35S::^GW^VYCE-pBar GATEWAY destination vectors containing the N- and C-terminal split parts of the enhanced YFP protein *Venus*, respectively [97]. BiFC constructs were transiently co-expressed by infiltration of *Agrobacterium tumefaciens* transformed with the corresponding binary vectors, and examined by confocal laser scanning microscopy (CLSM) 48 h after infiltration. For the development of U-box mutants, a DNA fragment between 709 and 739 bp (from ATG) on the U-box domain of *HvPUB15* was excised using *Bsa*XI and replaced by ligating synthetic oligos carrying the respective U-box mutation.

For co-immunoprecipitation (Co-IP), YFP-tagged HvARM1 and HvPUB15^ARM^ under the control of the 35S promoter were generated by cloning the full-coding sequence of HvARM1 (1–442 AA) or the ARM-repeat region of HvPUB15 (351–831 AA) into pEARLEYGATE104 (Earley et al., 2006). cMyc-Tagged HvThf1 (1–294 AA) and HvClpS1 (1–161 AA) under the control of the CaMV 35S promoter were generated by cloning into pGWB418 [98]. Mesophyll-protoplast transformation and co-immunoprecipitation was done as described [99].

### Subcellular localization of fluorescent proteins

For subcellular localization, full-length sequences of *HvPUB15*, *HvARM1,* *HvThf1*, and *HvClpS1* were N- and C-terminally fused in-frame to YFP in pIPKTA48 and pIPKTA49 vectors (Additional file 1: Figures S11 and S12). Resulting YFP-fusion constructs were transiently expressed in 7-day-old barley leaf segments by particle bombardment and examined after 12–24 h of incubation with or without *B. graminis* inoculation using CLSM.

### Genome-wide search for expressed partial gene duplicates

To identify expressed partial gene duplicates, a local database was generated using 23,614 full-length cDNA sequences of barley [100], and a Blast search was carried out against itself using the megablast tool in the Galaxy platform [101] with the recommended settings, except the percent identity cutoff was set to 80–99%. To exclude pairs of genes sharing only functional domains, an alignment-to-shorter-gene length ratio of at least 0.8 was set. To identify the open reading frames, a tBlastx analysis of the pairs was carried out using the Galaxy platform. Manual curation was done to exclude non-spliced transcripts and chimeric as well as partial clones. Identified full-length and partial genes were aligned using MAUVE and the MUSCLE algorithm (MegAlign Pro, Version 15.1.0 (155) DNASTAR).

## Additional files

Additional file 1: **Figure S1.** Asexual life cycle of *Blumeria graminis* and the assessment of fungal development on barley (*Hordeum vulgare*) and wheat (*Triticum aestivum*). **Figure S2.** Alignment of *HvPUB15* and *HvARM1* genomic sequences. **Figure S3.** Protein sequence alignment and *d*_N_/*d*_S_ analysis of ARM1 and PUB15. **Figure S4.** Off-target prediction in transgenic barley carrying the RNAi hairpin construct pIPKb009 for silencing of *HvARM1*. **Figure S5.** Expression of *HvARM1* endogenous transcripts in powdery mildew-attacked barley epidermis. **Figure S6.** Localization of YFP-tagged proteins in barley and *N. benthamiana* epidermal cells. **Figure S7.** In vitro ubiquitin ligase activity of HvPUB15. **Figure S8:** Additional controls for BiFC in *N. benthamiana* leaves. **Figure S9.** Quantification of BiFC signals in *N. benthamiana* leaves. **Figure S10.** Additional, array-based transcript regulation data of selected genes in the epidermis of powdery mildew-attacked barley leaves. **Figure S11.** Plasmid map of pIPKTA48 for the subcellular localization of N-terminal fusion proteins with YFP. **Figure S12.** Plasmid map of pIPKTA49 for the subcellular localization of C-terminal fusion proteins with YFP. **Figure S13.** Genome-wide analysis of expressed and conserved gene duplicates. **Figure S14.** Mutation of HvARM1 disrupts its resistance-enhancing function. **Table S8.** HvARM1-interacting candidates from yeast two-hybrid screening. **Table S9.** List of primers used in this study. **Table S10.** Sequences used for phylogenetic and *d*_N_/*d*_S_ analyses. (PDF 2000 kb)

Additional file 2: **Table S1. **Passport, phenotypic and gene haplotype data of barley accessions in the WHEALBI populations used for association mapping. **Table S2. **SNP calls and derived gene haplotypes for *HvARM1*. **Table S3. **SNP calls and derived gene haplotypes for *HvPUB15*. **Table S4. **Details of partial gene duplicate pairs in the barley genome. **Table S5. **Evolutionary conservation of partial gene duplicates in Triticeae tribe. **Table S6. **Transcript regulation of partial duplicates in host (Bgh) and non-host (Bgt) powdery mildew interaction in barley. **Table S7. **Primary microarray signal intensity data of *HvThf1* and *HvClpS1*. (XLSX 90 kb)

Additional file 3: Methods S1. Detailed description of materials and methods used in this study. (PDF 183 kb)
