# Supplementary material for: Evolutionarily conserved partial gene duplication in the Triticeae tribe of grasses confers pathogen resistance
Source: Genome Biol. 2018 Aug 15;19:116. doi: 10.1186/s13059-018-1472-7 (PMC6092874; doi:10.1186/s13059-018-1472-7)
Supplement: Supplementary file 1 — Figure S1. Asexual life cycle of Blumeria graminis and the assessment of fungal development on barley (Hordeum vulgare) and wheat (Triticum aestivum). Figure S2. Alignment of HvPUB15 and HvARM1 genomic sequences. Figure S3. Protein sequence alignment and dN/dS analysis of ARM1 and PUB15. Figure S4. Off-target prediction in transgenic barley carrying the RNAi hairpin construct pIPKb009 for silencing of HvARM1. Figure S5. Expression of HvARM1 endogenous transcripts in powdery mildew-attacked barley epidermis. Figure S6. Localization of YFP-tagged proteins in barley and N. benthamiana epidermal cells. Figure S7. In vitro ubiquitin ligase activity of HvPUB15. Figure S8: Additional controls for BiFC in N. benthamiana leaves. Figure S9. Quantification of BiFC signals in N. benthamiana leaves. Figure S10. Additional, array-based transcript regulation data of selected genes in the epidermis of powdery mildew-attacked barley leaves. Figure S11. Plasmid map of pIPKTA48 for the subcellular localization of N-terminal fusion proteins with YFP. Figure S12. Plasmid map of pIPKTA49 for the subcellular localization of C-terminal fusion proteins with YFP. Figure S13. Genome-wide analysis of expressed and conserved gene duplicates. Figure S14. Mutation of HvARM1 disrupts its resistance-enhancing function. Table S8. HvARM1-interacting candidates from yeast two-hybrid screening. Table S9. List of primers used in this study. Table S10. Sequences used for phylogenetic and dN/dS analyses. (PDF 2000 kb) [file 13059_2018_1472_MOESM1_ESM.pdf]

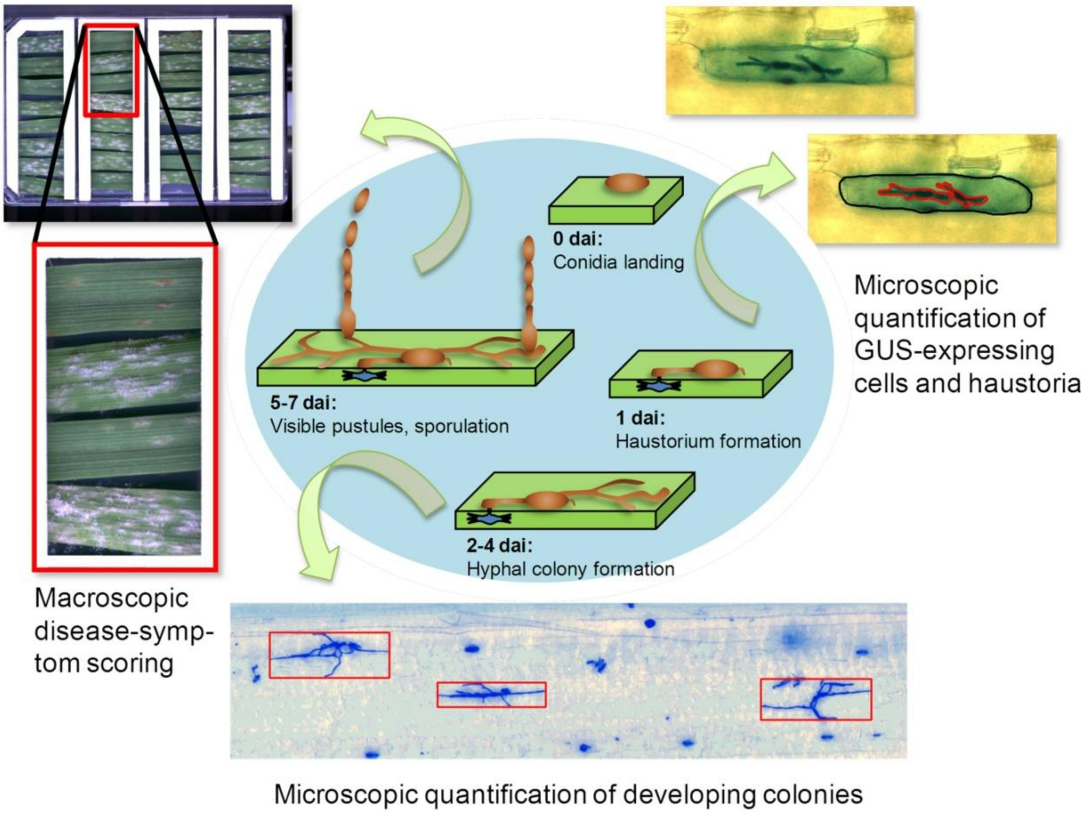

Figure S1: Asexual life cycle of *Blumeria graminis* and the assessment of fungal development in barley (*Hordeum vulgare*) and wheat (*Triticum aestivum*).

For transient-induced gene silencing (TIGS) and for transient over-expression (OEX), initial haustorium (feeding cell) formation in transformed, GUS-expressing epidermal cells of barley or wheat is completed 1 day after inoculation (dai) and was assessed under the microscope 40 h after inoculation. For the characterization of transgenic plants, early developing colonies were stained by Coomassie blue, counted and normalized to the analysed leaf area at 2 dai. The asexual life cycle is completed by the appearance of macroscopically visible, sporulating colonies (pustules) 5-7 dai, which can be estimated or quantified as percentage of pustule-covered leaf area. However, for the results described here, macroscopic disease quantification was not used.

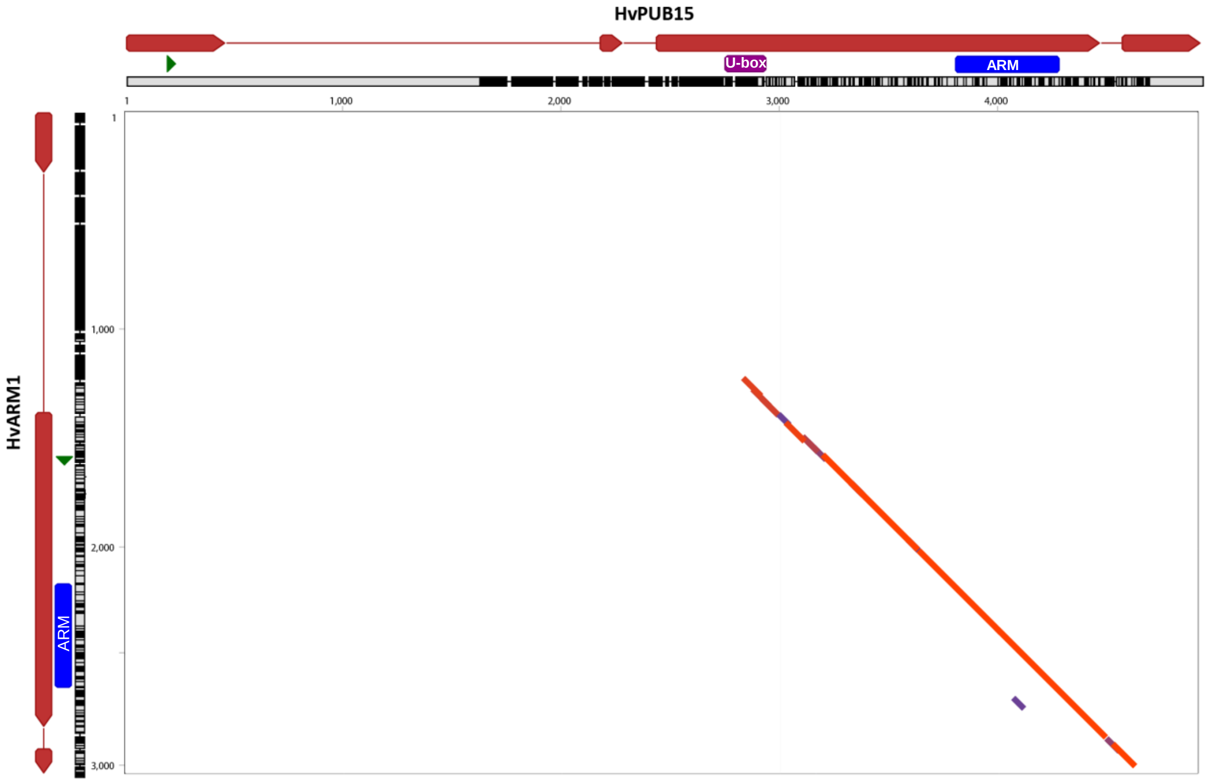

Figure S2: Alignment of *HvPUB15* and *HvARM1* genomic sequences.
Dot plot alignment showing the similarity between the genomic sequences of *HvPUB15* (on the top) and *HvARM1* (on the Y-axis). The dot matrix chart was generated using Geneious 10.0.9 [1] with high sensitivity/fast setting using a window size of 30, threshold of 80 and tile size of 1000. Dot plot lines are generated by the software according to the length of the match: blue for short matches and red for matches over 100bp. U-box domain and ARM repeat domain (ARM) are indicated on *HvPUB15* and *HvARM1,* respectively. Green arrowheads indicate start codon position.

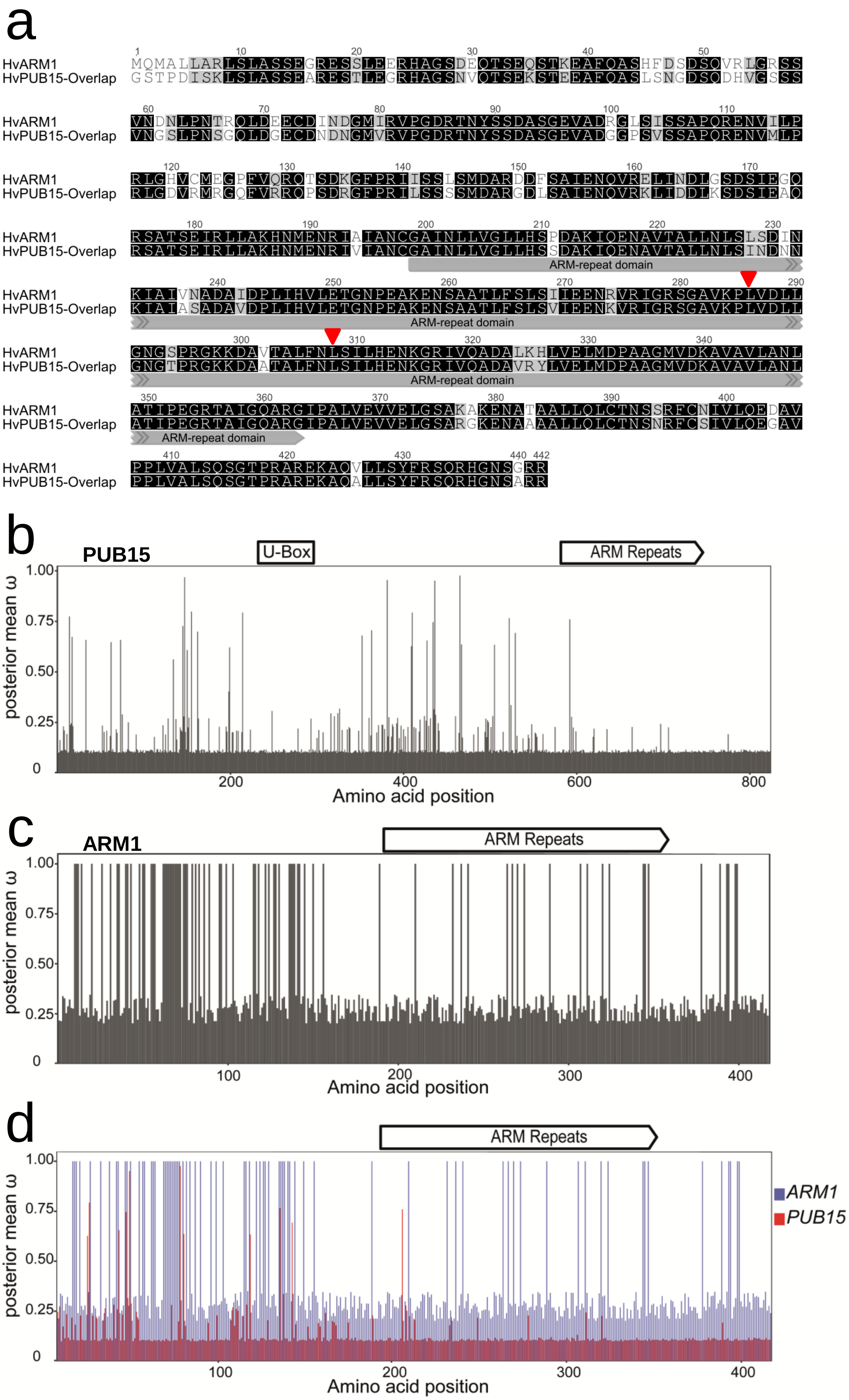
a
HvARM1 MQMALLARLSLASSEGRESSLEERHAGSDEQTSEQSTKEAFQASHFDSDSQVRLGRSS
HvPUB15-Overlap GSTPDISKLSLASSEARESTLEGRHAGSNVQTSEKSTEEAFQASLSNGDSQDHVGSSS
HvARM1 VNDNLPNTRQLDEECDINDGMIRVPGDRTNYSSDASGEVADRGLSISSAPQRENVILP
HvPUB15-Overlap VNGSLPNSGQLDGECDNDNGMVRVPGDRTNYSSDASGEVADGGPSVSSAPQRENVMLP
HvARM1 RLGHVCMEGPFVQRQTSDKGFPRIISSLSMDARDDFSAIENQVRELINDLGSDSIEGQ
HvPUB15-Overlap RLGDVRMRGQFVRRQPSDRGFPRILSSSSMDARGDLSAIENQVRKLIDDLKSDSIEAQ
HvARM1 RSATSEIRLLAKHNMENRIAIANCGAINLLVGLLHSPDAKIQENAVTALLNLSLSDIN
HvPUB15-Overlap RSATSEIRLLAKHNMENRIVIANCGAINLLVGLLHSSDAKIQENAVTALLNLSINDNN
ARM-repeat domain
HvARM1 KIAIVNADAIDPLIHVLETGNPEAKENSAATLFSLSIIEENRVRIGRSGAVKPLVDLL
HvPUB15-Overlap KIAIASADAVDPLIHVLETGNPEAKENSAATLFSLSVIEENKVRIGRSGAVKPLVDLL
ARM-repeat domain
HvARM1 GNGSPRGKKDAVTALFNLSILHENKGRIVQADALKHLVELMDPAAGMVDKAVAVLANL
HvPUB15-Overlap GNGTPRGKKDAATALFNLSILHENKGRIVQADAVRYLVELMDPAAGMVDKAVAVLANL
ARM-repeat domain
HvARM1 ATIPEGRTAIGQARGIPALVEVVELGSAKAKENATAALLQLCTNSSRFCNIVLQEDAV
HvPUB15-Overlap ATIPEGRTAIGQARGIPALVEVVELGSARGKENAAAALLQLCTNSNRFCSIVLQEGAV
ARM-repeat domain
HvARM1 PPLVALSQSGTPRAREKAQVLLSYFRSQRHGNSGRR
HvPUB15-Overlap PPLVALSQSGTPRAREKAQALLSYFRSQRHGNSARR
b
PUB15
U-Box
ARM Repeats
posterior mean ω
Amino acid position
c
ARM1
ARM Repeats
posterior mean ω
Amino acid position
d
ARM Repeats
posterior mean ω
Amino acid position
ARM1
PUB15

Figure S3: Protein sequences alignment and d_N/d_S analysis of ARM1 and PUB15

(a) Alignment of amino-acid sequences of HvARM1 (starting at AA1) and HvPUB15 (starting at AA390). ARM repeat domain is shown in grey shade under the alignment; Red inverted triangle, sites of point mutation (Additional file 1: Figure S14). Pattern of selection pressure detected along PUB15 **(b)** and ARM1 **(c)** genes, respectively and among the overlapping region of PUB15 and ARM1 genes in the selected six Triticeae species plus rice **(d)** as determined by the ratio of non-synonymous to synonymous nucleotide exchanges (ω) per codon. An ω < 1 suggests constraint, or purifying selection while an ω > 1 indicates positive selection and ω = 1 for neutral changes. Position of U-box and ARM repeat domain is indicated.

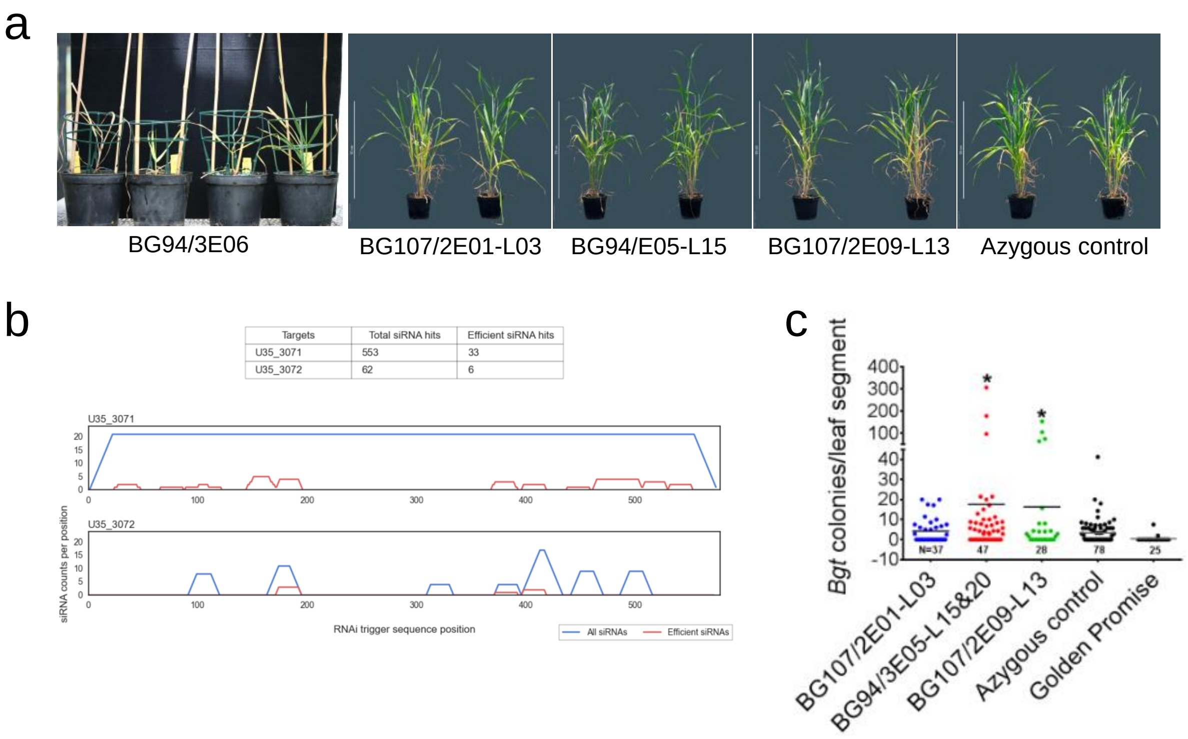

Figure S4: (**a**) Growth phenotypes of *HvARM1*-RNAi transgenic seedlings from event BG94/3E06 exhibiting more severe seedling lethality in T1 (not used for phenotyping in T3 generation) and of adult plants from event BG107/2E01, BG94/E05, and BG107/2E09 in T3 that was used for *Bgh* and *Bgt* resistance tests. (**b**) Prediction by the si-Fi21 software of siRNA hits in target-transcript Hv*ARM1* (U35_3071) and potential off-target Hv*PUB15* (U35_3072) by using the hairpin dsRNA region of the construct for stable RNAi plants as input sequence. The search was conducted in the "off-target prediction" mode of si-Fi21, including default setting of end stability difference and target-site accessibility. **(c)** Detached second leaves of T3 transgenic barley RNAi plants were inoculated with *Bgt* and infection was assessed microscopically 72 hours after inoculation. Data represent normalized colony density (number/cm^2/leaf area) from 2-3 biological replications and horizontal line indicate the mean. Statistical differences between transgenic events and azygous plants are indicated by asterisks. *, $p<0.05$ (student's t-test; two-tailed).

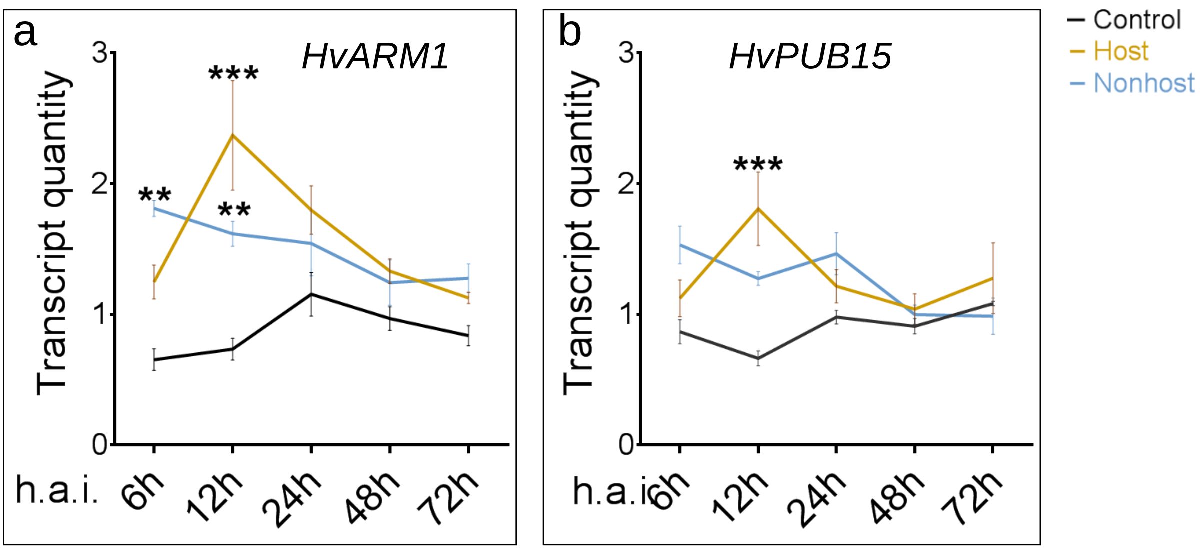

Figure S5: Expression of *HvARM1* in barley leaf epidermis following *B. graminis* attack. Transcript quantities of *HvARM1* and Hv*PUB15* were determined by RT-qPCR in RNA samples of epidermal peels using TaqMan probes. Mean ± SD of transcript quantity (relative to Hv*UBC,* Acc. AK252586.1) from two biological replicates with three technical replicates each are shown. Differences between control and inoculated samples are indicated by asterisks. **, p<0.005; ***, p<0.0005 (ANOVA with Bonferroni's multiple comparisons test).

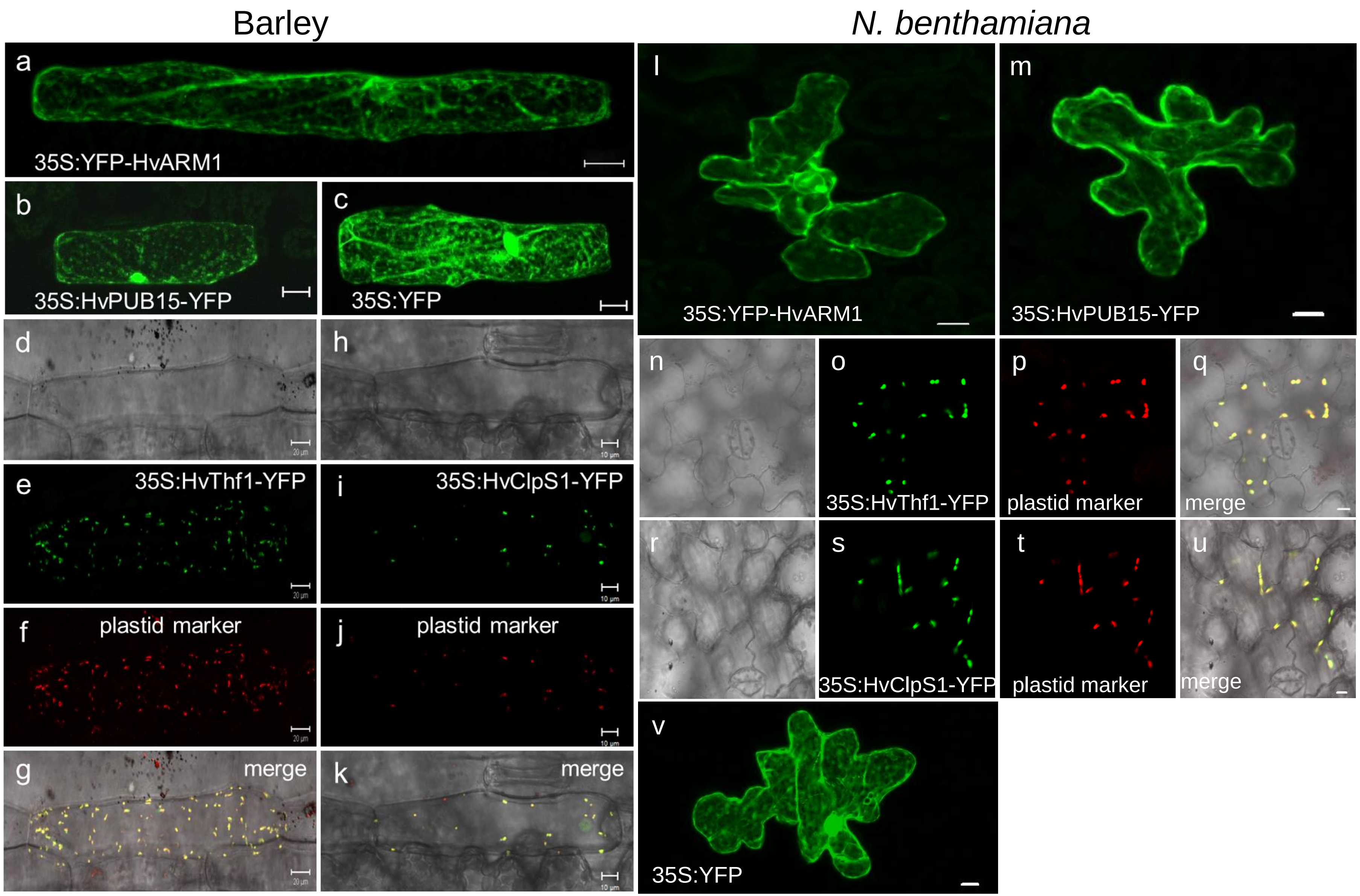
Barley
N. benthamiana
a
35S:YFP-HvARM1
b
35S:HvPUB15-YFP
c
35S:YFP
d
h
e
35S:HvThf1-YFP
i
35S:HvClpS1-YFP
f
plastid marker
j
plastid marker
g
merge
k
merge
l
35S:YFP-HvARM1
m
35S:HvPUB15-YFP
n
o
35S:HvThf1-YFP
p
plastid marker
q
merge
r
s
35S:HvClpS1-YFP
t
plastid marker
u
merge
v
35S:YFP

Figure S6: HvARM1 and HvPUB15 are localized in the cytoplasm whereas two putative interacting proteins are targeted to plastids.

Fluorescently labelled fusion proteins were transiently expressed in barley and *N. benthamiana* epidermal cells by particle bombardment. Micrographs were taken 12-18 h after bombardment by using a confocal laser-scanning microscope. The images shown are representative samples from four biological replications in barley and two in *N. benthamiana*. Panels (a and b, l and m), localization of HvARM1 fused N-terminally to YFP, HvPUB15 fused C-terminally to YFP in barley and *N. benthamiana*, respectively. Panel (c) localisation of wildtype YFP in barley. Panels (d and h, and n and r), bright field images, panels (e and i, and o and s), punctate localization of HvThf1 and HvClpS1 fused C-terminally to YFP in barley and *N. benthamiana,* respectively. Panels (f and j, and p and t), localization of the plastid marker obtained by fusing mCherry N-terminally to the first 79 amino acids of Rubisco small subunit (SSU, construct pt-rkCD3-999; [2]). Panels (g and k, and q and u), merge of bright field, YFP- and mCherry signals from panels (d-f), (h-j), (n-p) and (r-t) in barley and *N. benthamiana,* respectively. Panel (c and v) localisation of wildtype YFP in barley and benthamiana,respectively.

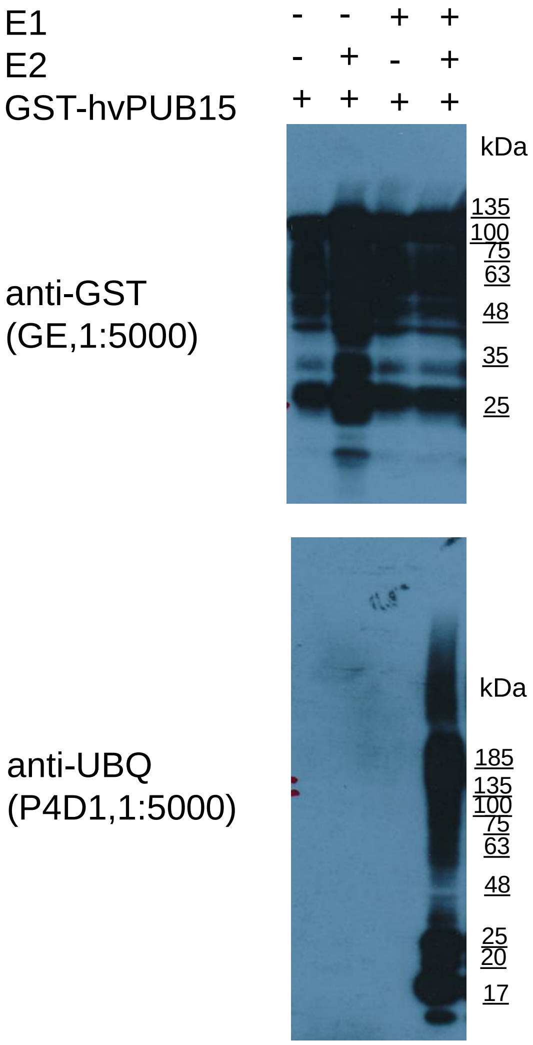

Figure S7: In vitro ubiquitin ligase activity of HvPUB15.

In vitro ubiquitination assay was performed as described [3] with the following modifications: Recombinant proteins were expressed by 1:50 transfer of an overnight culture of *E.coli* Rosetta 2 cells (expression vector pGEX4T1) into 100 ml LB medium (without antiobitics) and addition of 0.1 mM IPTG for 3 h at OD=0.5 (28°C). Proteins were purified (in 15 ml Sarstedt tubes) by sonication at 50% 3x 15 sec in 10 ml PBS (AEBSF, 5mM DTT), followed by 40 min 100,000 x g centrifugation. 25 µl of slurry was incubated for 1h at RT, followed by washing 3 times with PBS (+AEBSF, DTT) and centrifugation up to 500g. Aliquots of the reactions were separated by SDS-PAGE, followed by protein blotting and immunodetection of ubiquitinated proteins or ubiquitin oligomers.

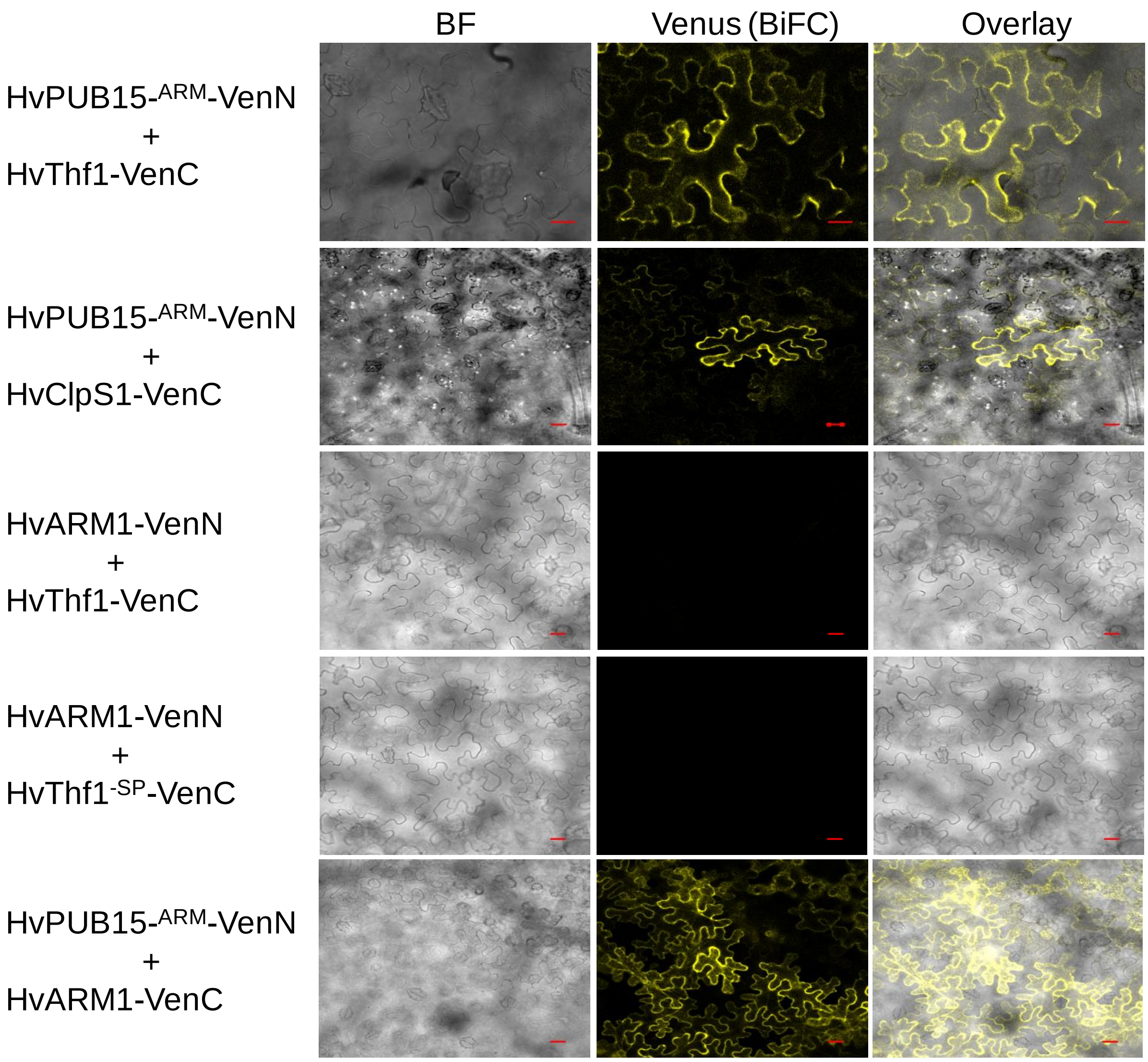

Figure S8: Additional controls for BiFC in *N. benthamiana* leaves.

The specificity of HvPUB15 interaction with HvThf1 and HvClpS1 was further verified using only the ARM-domain of HvPUB15. A BiFC signal indicates that ARM-domain of HvPUB15 is involved in interaction with HvThf1 and HvClpS1. Further, the specificity of HvARM1 interaction with HvThf1 was confirmed by switching the split-YFP parts and also by deletion of N-terminal plastid import signal (HvThf1^{-SP}). Absence of BiFC signal in HvARM1-VenN+HvThf1-VenC and HvARM1-VenN +HvThf1^{-SP}-VenC and presence of BiFC signal in HvThf1^{-SP}-VenN+HvARM1-VenC (Figure 4) indicates specificity in interaction. BF, bright field; -SP, with deleted N-terminal plastid import signal; VenN, N-terminal half of the stabilized YFP version “Venus”; VenC, C-terminal half of the YFP “Venus”. Scale bars, 20 µm.

HvPUB15 + HvThf1 BiFC

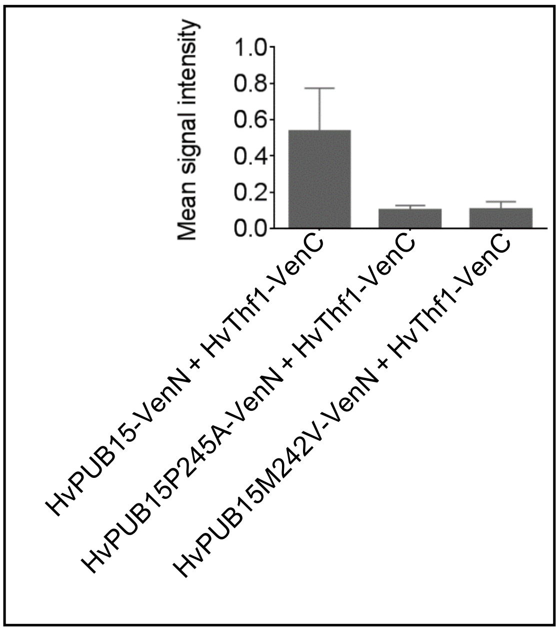

HvPUB15 + HvClpS1 BiFC

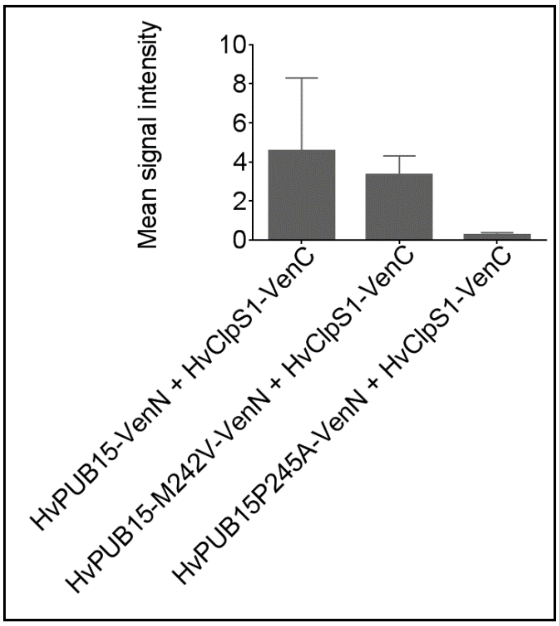

HvARM1 + HvThf1 and HvARM1 + HvClpS1 BiFC

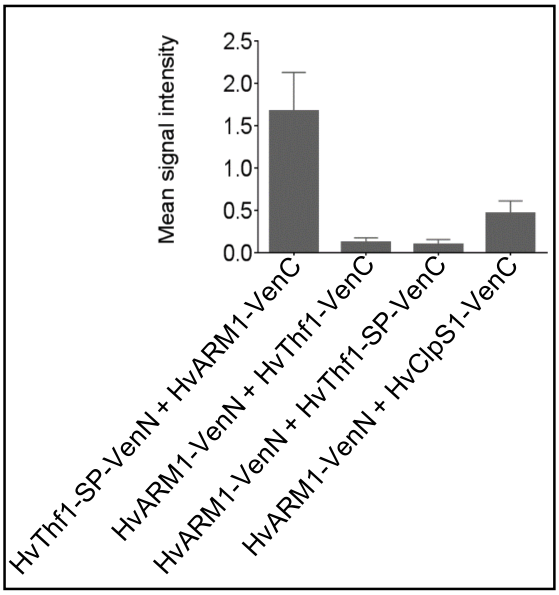

HvPUB15 + HvARM1 BiFC

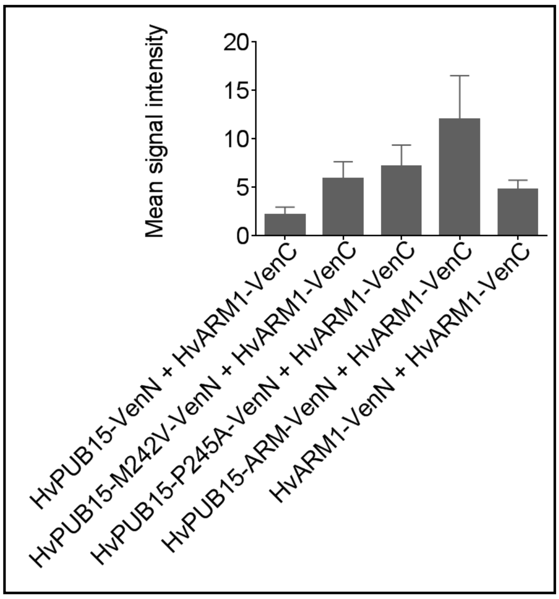

Figure S9: Quantification of BiFC signals in *N. benthamiana* leaves.
In order to quantify the specificity of BiFC intensity signal, approximately 10 images per interaction were acquired at random positions on infiltrated leaf areas with constant imaging settings [4]. Mean YFP-signal intensity was calculated using the ZEN software (ver.2.1). Data represents the mean ± SD.

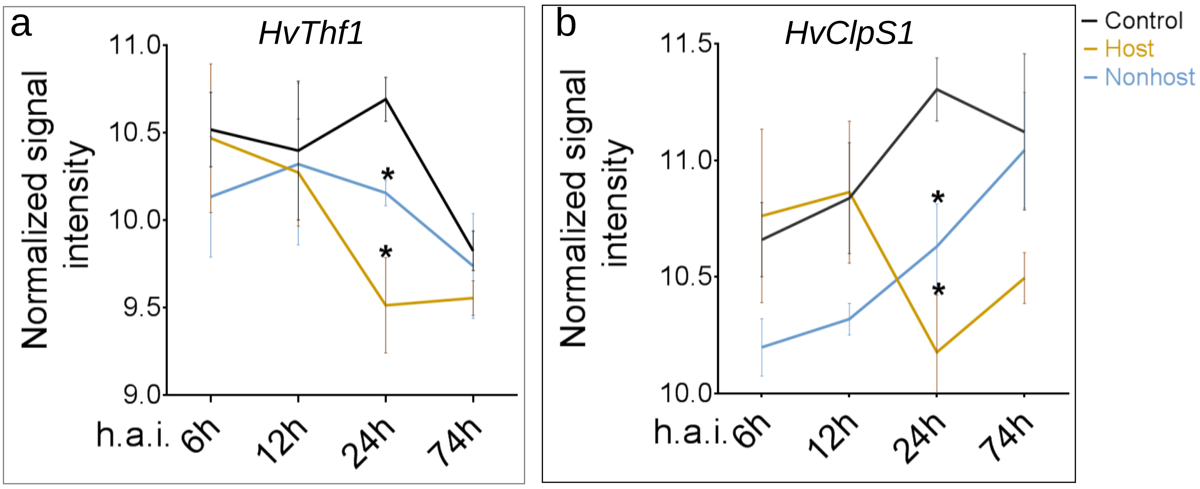

Figure S10: Time course of Hv*Thf1* (a) and Hv*ClpS1* (b) mRNA levels in barley epidermal peels challenged with host (*Bgh*) and nonhost (*Bgt*) powdery mildew pathogens. Profile plots show quantile-normalized signal intensity values (log_2 transformed) from the Agilent 44k oligonucleotide array [5]. Mean ± SEM of 3 biological replicates; * indicates significant differences from non-inoculated control at $p<0.05$ level (unpaired t-test unpaired, two-tailed). Normalized signal intensities of the two transcripts are shown in Additional file 2: Table S7.

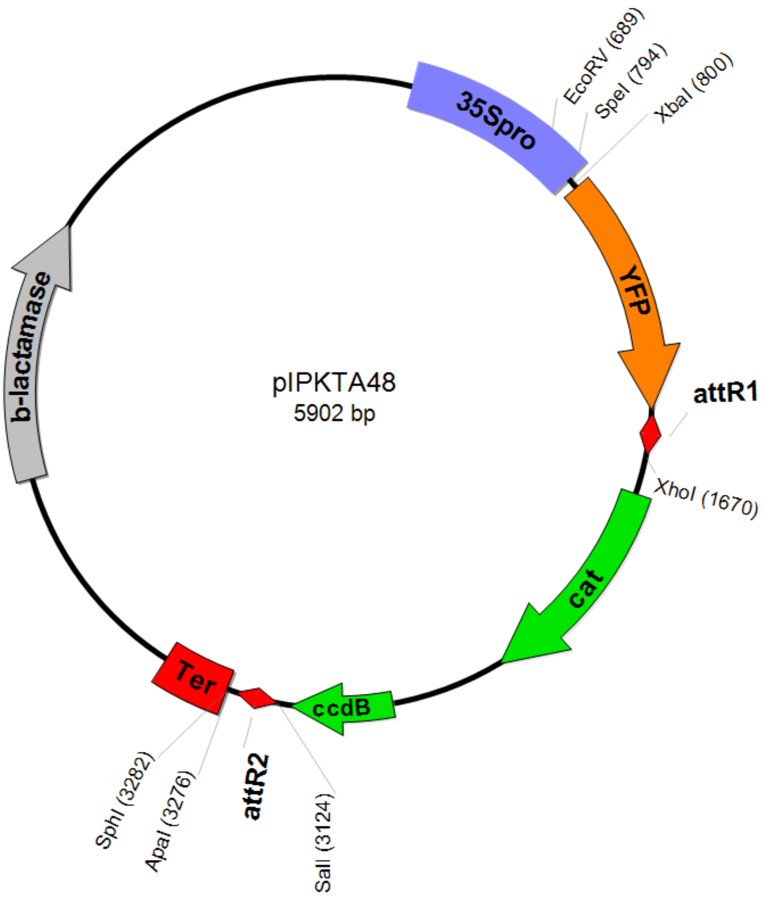

Figure S11: Plasmid map of pIPKTA48 for transient expression of YFP-tagged plant proteins.

The transient expression vector pIPKTA9 [6] was double-digested with *Xho*I, and *Xba*I. The gateway cassette including the YFP tag from the binary vector pGWB41 [7] was double-digested with *Xba*I-*Eco*ICRI (*Ecl*136II) and ligated into pIPKTA9 between the *Xho*I-*Xba*I sites. This resulted in the Gateway-compatible transient expression vector pIPKTA48 to allow N-terminal YFP fusions.

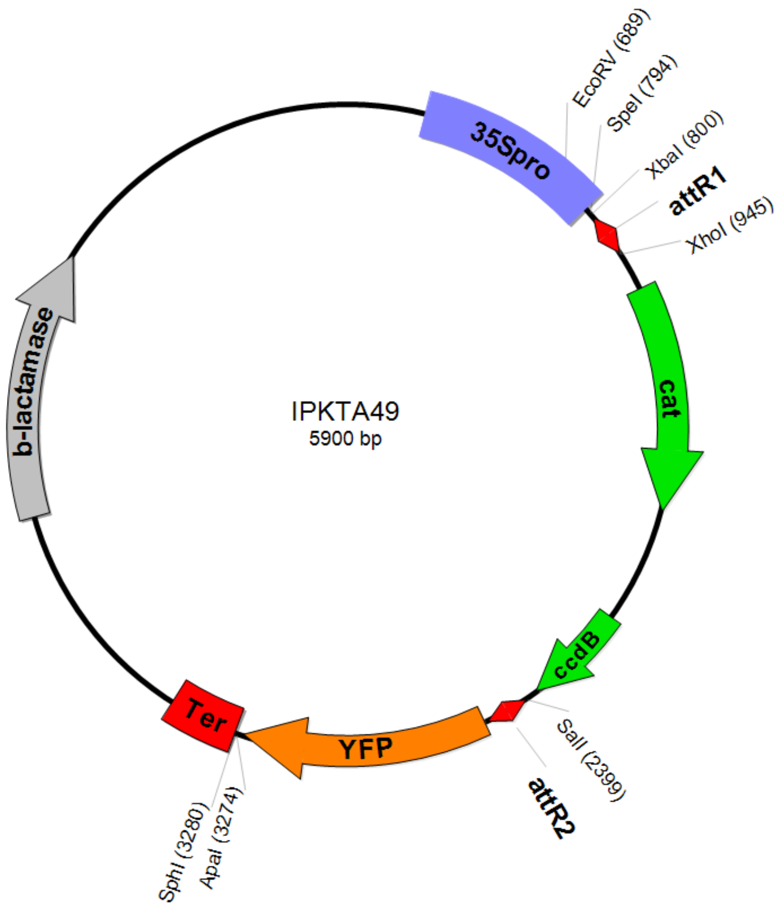

Figure S12: Plasmid map of pIPKTA49 for transient expression of YFP-tagged plant proteins.

The transient expression vector pIPKTA9 [6] was double-digested with *Xho*I, and *Xba*I. The gateway cassette including the YFP tag from the binary vector pGWB42 [7] was double-digested with *Xba*I-*Eco*ICRI (*Ecl*136II) and ligated into pIPKTA9 between the *Xho*I-*Xba*I sites. This resulted in the Gateway-compatible transient expression vector pIPKTA49 to allow C-terminal YFP fusions.

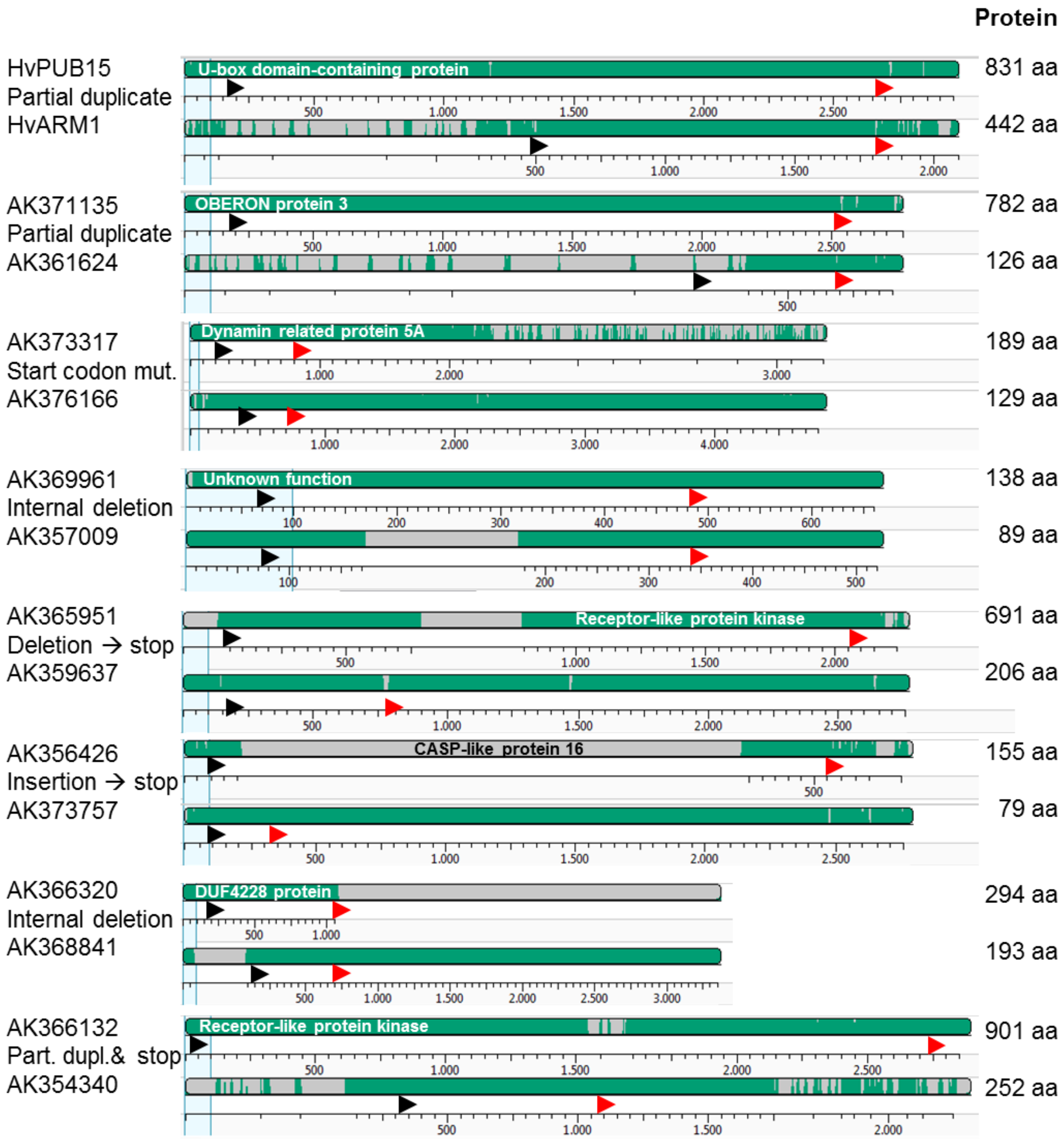

Figure S13: Genome-wide analysis of expressed and conserved gene duplicates.

Pairs of barley cDNA encoding full-length proteins and partial copies, respectively, were aligned using MUSCLE algorithm. The dark green shadings are aligning sequences whereas grey shadings show gaps. Black and red arrowheads indicate start- and stop-codon positions of the corresponding open reading frames, respectively. Gene IDs and resulting short ORF is given on the left and predicted amino acid (aa) length is given on the right. Annotation and further details can be found in the Additional file 2: Table S4.

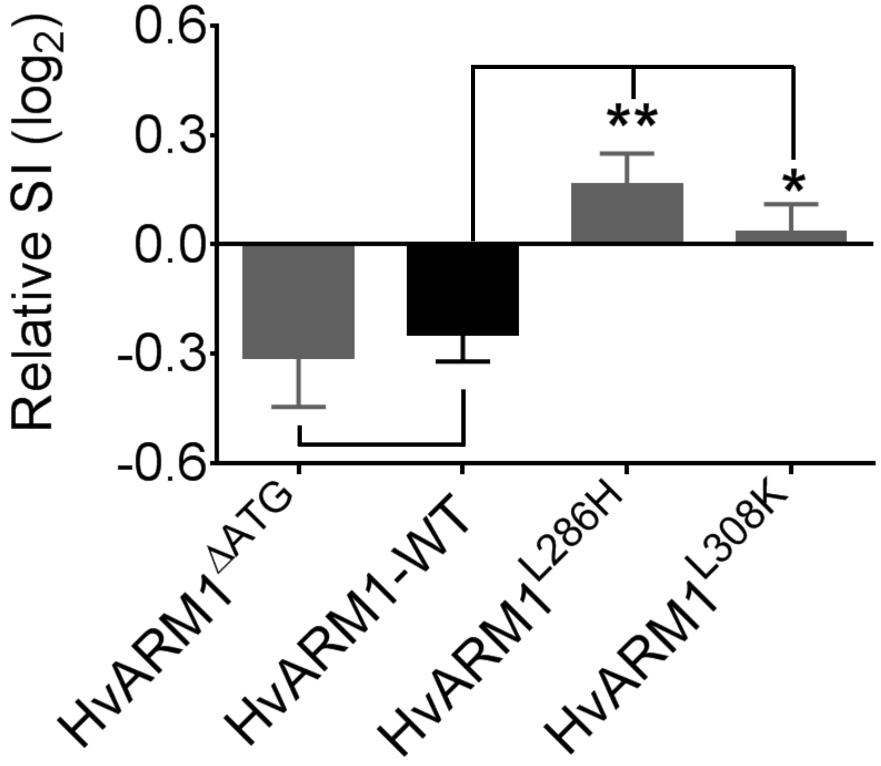

Figure S14: Mutation of HvARM1 disrupts its resistance-enhancing function.

HvARM1-WT (wildtype) or mutant versions without the translation-initiation codon (*HvARM1*$^{\Delta ATG}$) or with single amino-acid exchange (*HvARM1*^{L286H} and *HvARM1*^{L308K}) were transiently expressed in barley epidermal cells of cv. Maythorpe, followed by *Bgh* inoculation 4 h after bombardment. Mean ± SE from 5-6 independent experiments of the relative susceptibility index (SI), normalized to the internal empty-vector control of each experiment, are shown. Normalized HvARM1-WT SI values compared against mutant versions (unpaired t-test; 1-tailed). Statistical differences are indicated by asterisks. *, $p<0.05$; **, $p<0.005$. Note that statistical significance of HvARM1-WT (SI values compared against the hypothetical value "0"; One-sample t-test; 1-tailed; *, $p<0.05$) is not indicated for easy understanding. Mutated amino acid positions are indicated in Additional file 1: Figure S3-a. Amino acid code: L-Leucine; H-Histidine; K-Lysine.

Table S8: Description of HvARM1-interacting proteins.

Barley high-confidence gene ID	Description in bex-DB[a]	Full-length cDNA clone ID NIAS[b]	BlastX description (NCBI) of most significant hit	InterProScan domain description[c]
HORVU3Hr1G117760	DNAj type protein	FLbaf6n23	DnaJ homolog subfamily C member 3 [Triticum urartu]	Tetratricopeptide repeat-containing domain
HORVU2Hr1G041260	ToxA binding protein	NIASHv1005F13	Chloroplast-localized Ptr ToxA-binding protein1 [Triticum aestivum]	Protein Thf1
HORVU7Hr1G020580	Cadmium tolerant	NIASHv3024H04	hypothetical protein TRIUR3_00244 [Triticum urartu]	None predicted
HORVU2Hr1G080670	Microtubule associated complex	NIASHv3078K10	putative dynein light chain type 1 family protein [Zea mays]	Dynein light chain superfamily
HORVU3Hr1G059130	Protein serine/threonine kinase activity	NIASHv1006F21	protein kinase domain containing protein [Zea mays]	Protein kinase-like domain superfamily
HORVU2Hr1G003460	ATP-Dependent Clp protease adaptor	FLbaf7l02	ATP-dependent Clp protease adapter protein ClpS [Aegilops tauschii]	Adaptor protein ClpS, core

[a]http://www.plexdb.org/plex.php?database=Barley
[b]http://barleyflc.dna.affrc.go.jp/bexdb/
[c]http://www.ebi.ac.uk/interpro/

Table S9: PCR primers used in this study.

Gene or Construct	Primer	Sequence 5' to 3'	Tm	Used for
**HvARM1 TaqMan**	Forward<br>Reverse<br>Probe	TCTCAGATGATAGTTATTGTTGTTG<br>TAAACCCGAAGAATACACCA<br>6FAM-TTGCTGTGTTTGTGACTGGTCG-BHQ1	58	Transcript quantification
**HvPUB15 TaqMan**	Forward<br>Reverse<br>Probe	CGGTTAAATTTGTTGACTGG<br>AACCGACAAAATACACCATC<br>6FAM-TGAGTGTGGGCGTTGGTTGT-BHQ1	58	Transcript quantification
**HvUBC2 TaqMan**	Forward<br>Reverse<br>Probe	ACTCCGAAGCAGCCAGAATG<br>GATCAAGCACAGGGACACAAC<br>Yakima yellow-AGAACAAGCGCGAGTACAACCGCAAGGTG-BHQ1	58	Transcript quantification
**pIPKTA30_HvARM1**	Froward<br>Reverse	CTCTTTCCCGTGCATTTCC<br>AGATCATACCTCAGAAACCAATGG	61	TIGS
**pIPKTA30_HvPUB15**	Froward<br>Reverse	GGAATTGAAACGGTCCACC<br>ACCTCCTTCGCCAGATCAA	55	TIGS
**pGBKT7-HvARM1 (bait)**	Froward<br>Reverse	GTCTTTAGCATATGCAAATGGCTCTGCTAG<br>ACCATCGTCGACATCTCCTTCCCGA	60	Y2H
**pGBKT7-HvPUB15 (bait)**	Froward<br>Reverse	TGATCCTCATATGGAAGATTTCTC<br>TGCTAACATCATCATCATAATCATC	58	Y2H
**pEARLYGATE104-HvARM1**	Forward<br>reverse	ggggACAAGTTTGTACAAAAAAGCAGGCTTCATGCAAATGGCTCTGCTAGCAAGGC<br>ggggACCACTTTGTACAAGAAAGCTGGGTTTTATCCTCATCTCCTTCCCGAGTTCC	58	BiFC
**pEARLYGATE104-HvPUB15 -ARM domain**	Forward<br>reverse	ggggACAAGTTTGTACAAAAAAGCAGGCTTCATGAAAAGCCTGAACAGAGCA<br>ggggACCACTTTGTACAAGAAAGCTGGGTTCATCATCTCCTCGCCGAGTTCC	58	BiFC

**pGWB418-HvThf1**	Forward	ggggACAAGTTTGTACAAAAAAGCAGGCTTCATGGCGGCCATATCGTCG	60	BiFC
	reverse	ggggACCACTTTGTACAAGAAAGCTGGGTATGCTTCAAGGGATAAGTGCTTC		
**pGWB418-HvClpS1**	Forward	ggggACAAGTTTGTACAAAAAAGCAGGCTtcATGCTGCCAGGATGCTC	60	BiFC
	reverse	ggggACCACTTTGTACAAGAAAGCTGGGTCTATATGGGATGATATGTGTAAA		
**pQE30-HvARM1**	Forward	CGGGATCCATGCAAATGGCTCTGCT	60	BiFC
	reverse	GCTGCAGTCATCTCCTTCCCGAGTT		
**pGEX4T1-HvPUB15**	Forward	CGGGATCCATGGAAGATTTCTCACC	60	BiFC
	reverse	AAGCGGCCGCTCATCTCCTCGCCGA		
**U35_19170 (DNAj)**	Froward	AACCAAGGAAGGAAGGAAGG	58	TIGS
	Reverse	CATTTGAATAGTCTTTCAGTGCTAGC		
**U35_374 (ToxABP1)**	Froward	CTTCCAGAGATGGGGAAATAGA	58	TIGS
	Reverse	AAATTACAAAATACCAACTACTGCGA		
**HvARM1-S229G***	Froward	CCCTCCTTAATTTGTCACTC**G**GTGATATCAATAAGATTGCC	55	OEX
	Reverse	CACGATGGCAATCTTATTGATATCAC**C**GAGTGACAAATTAAGG		
**HvARM1-L286H***	Froward	GTGCTGTAAAGCCTC**A**CGTGGACTTGCTGGG	55	OEX
	Reverse	CCCATTTCCCAGCAAGTCCACG**T**GAGGCTTTAC		
**HvARM1-L308K***	Froward	AGAAAGATGCGGTTACTGCATTGTTTAAT**AA**ATCCATACTTCATGAGAAA	55	OEX
	Reverse	CCCTTGTTCTCATGAAGTATGGAT**TT**ATTAAACAATGCAGTAACCGC		
**HvPUB15-M242V***	Forward	TTTCCCTGGAGCTG**G**TGTCTGATCCTGTTA	60	BiFC
	reverse	AAAGGGACCTCGAC**C**ACAGACTAGGAC		
**HvPUB15-P245A***	Forward	TTTCCCTGGAGCTGATGTCTGAT**G**CTGTTA	60	BiFC
	reverse	AAAGGGACCTCGACTACAGACTA**C**GAC		
**U35_1321 (Cd.tol)**	Froward	ATGGAGGCCAGTGAATTCCACC	58	TIGS
	Reverse	AGCAGCAGCAGAGCCAGTCG		

**U35_23261 (Mic.tub)**	Froward<br>Reverse	GAGGCCAGTGAATTCCACCCAAG<br>ATTTGCGCTAAATCTACACTTG	58	TIGS
**U35_22515 (kinase)**	Froward<br>Reverse	ATGAAGGAGCTGCCAGCTAGCC<br>ACTATGTACACCTGCTAAAAATG	58	TIGS
**U35_829 (Clp-S1)**	Froward<br>Reverse	ATGCTGCCAGGATGCTC<br>TCAGCAGCCACCACTTGCAGG	58	TIGS
**U35_374 (ToxABP1)**	Froward<br>Reverse	TGCTCTAGAGACTCTCCCATGGCGG<br>TGCTCTAGAGCGTTGAGTTAATGCTTC	58	OEX
**U35_829 (Clp-S1)**	Froward<br>Reverse	TGCTCTAGAGCCTCCTCCCCGGCAGC<br>GCTCTAGAGCTCAGCAGCCACCACTTGC	55	OEX
**pIPKTA48-YFP-HvARM1 (N-ter fusion)**	Froward<br>Reverse	ggggACAAGTTTGTACAAAAAAGCAGGCTTCATGCAAATGGCTCTGCTAGCAAGGC<br>ggggACCACTTTGTACAAGAAAGCTGGGTTTTATCCTCATCTCCTTCCCGAGTTCC	58	Cellular localization
**pIPKTA49-YFP-HvPUB15 (C-ter fusion)**	Froward<br>Reverse	ggggACAAGTTTGTACAAAAAAGCAGGCTATGGAAGATTTCTCACCGAGGGC<br>ggggACCACTTTGTACAAGAAAGCTGGGTGTCTCCTCGCCGAGTTCCCGT	58	Cellular localization
**pIPKTA49-YFP-HvThf1(C-ter fusion)**	Froward<br>Reverse	ggggACAAGTTTGTACAAAAAAGCAGGCTTATGGCGGCCATATCGTCGC<br>ggggACCACTTTGTACAAGAAAGCTGGGTGATGCTTCAAGGGATAAGTGCTT	60	Cellular localization
**pIPKTA49-YFP-HvClp-S1 (C-ter fusion)**	Froward<br>Reverse	ggggACAAGTTTGTACAAAAAAGCAGGCTATGCTGCCAGGATGCTC<br>ggggACCACTTTGTACAAGAAAGCTGGGTGGCAGCCACCACTTGCAGGTT	60	Cellular localization

**HptII**	Forward<br>reverse	AGCTGCGCCGATGGTTTCTACAA<br>CATCGCCTCGCTCCAGTCAATG	58	RNAi plants screening
**RNAi repeat I**	Forward<br>reverse	GGAGAGGACACGCTGAAATC<br>AGGCAACTAGATAACCATTTTCA	58	RNAi plants screening
**RNAi repeat II**	Forward<br>reverse	ATGAGCGAAACCCTATAAGAACCCTA<br>GGATAGCCCTCATAGATAGAGTACTAACTAA	58	RNAi plants screening
**35Spro**	Forward<br>reverse	GTGATTTCAGCGTGTCCTCT<br>CAA CCA CGT CTT CAA AGC AA	58	RNAi plants screening
**HvGAPDH**	Forward<br>reverse	CTAGCAGCCCTTCCACCTCTCCA<br>CAATGCTAGCTGCACCACCAACTG	58	RNAi plants screening

*- Mutated site is shown in bold font

Table S10: Sequences used for phylogenetic and d_N/d_S analyses

sequence name	genomic	transcripts	protein
OsPUB15	NW_015379181.1:551574-556921[a]	XM_015795011[a]	XP_015650497[a]
HvPUB15	chr3H:689573847-689580280[b]	HORVU3Hr1G113910.3	HORVU3Hr1G113910
HvARM1	chr3H:594730498-594734223[b]	HORVU3Hr1G081380.4	HORVU3Hr1G081380
ScPUB15	Lo7_v2_scaffold_446234:9254-16180[c]	KM881629[a]	AJN91234[a]
ScARM1	Lo7_v2_scaffold_169416:20330-24274[c]	KM881628[a]	AJL33746[a]
AesPub15	TGAC_WGS_speltoides_v1_contig_203746:1-2698<br>TGAC_WGS_speltoides_v1_contig_252331:1-4357[d]	inferred[g]	inferred[g]
AesARM1	TGAC_WGS_speltoides_v1_contig_481548:2394-5169[d]	inferred[g]	inferred[g]
AetPUB15	NW_017952709.1:1885887-1893057[a]	XM_020311842[a]	XP_020167431[a]
AetARM1	NW_017937549.1:3945-7078[a]	XM_020296982[a]	XP_020152571[a]
TuPUB15	TGAC_WGS_urartu_v1_contig_1357654:1432-6837[d]	TRIUR3_31889-T1[e]	EMS61710[a]
TuARM1	TGAC_WGS_urartu_v1_contig_181472:955-3868[d]	TRIUR3_13873-T1[e]	EMS55038[a]
TaA-PUB15	TGACv1_scaffold_195661_3AL:30237-37497[e]	AA0652480[e]	A0A1D5VCC0[f]
TaA-ARM1	TGACv1_scaffold_196567_3AL:29997-33195[e]	AA0661500[e]	W5CA56[f]
TaB-PUB15	TGACv1_scaffold_222817_3B:15836-23050[e]	AA0771270[e]	A0A077RF03[f]
TaB-ARM1	TGACv1_scaffold_223941_3B:27077-30083[e]	AA0789920[e]	A0A0H2UIP0[f]
TaD-PUB15	TGACv1_scaffold_249335_3DL:122337-128710[e]	AA0845740[e]	A0A1D5WE99[f]
TaD-ARM1	TGACv1_scaffold_249751_3DL:39809-43090[e]	AA0855640[e]	W5DDJ6[f]

a: NCBI

b: http://webblast.ipk-gatersleben.de/barley_ibsc

c: Schmutzer T: Scaffolds of rye (Secale cereale L.) inbred line Lo7– version 2. https://doi.org/10.5447/ipk/2016/56

d: https://urgi.versailles.inra.fr/blast/blast.php

e: http://ensembl.gramene.org/Tools/Blast?db=core

f: UniprotKB

g: no transcripts or proteins directly available, open reading frames inferred by homology to the other sequences and especially the wheat B transcripts and translated in Geneious

1. Kearse M, Moir R, Wilson A, Stones-Havas S, Cheung M, Sturrock S, Buxton S, Cooper A, Markowitz S, Duran C, et al: **Geneious Basic: an integrated and extendable desktop software platform for the organization and analysis of sequence data.** *Bioinformatics* 2012, **28:**1647-1649.
2. Nelson BK, Cai X, Nebenfuhr A: **A multicolored set of in vivo organelle markers for co-localization studies in Arabidopsis and other plants.** *Plant J* 2007, **51:**1126-1136.
3. Stegmann M, Anderson RG, Ichimura K, Pecenkova T, Reuter P, Zarsky V, McDowell JM, Shirasu K, Trujillo M: **The ubiquitin ligase PUB22 targets a subunit of the exocyst complex required for PAMP-triggered responses in Arabidopsis.** *Plant Cell* 2012, **24:**4703-4716.
4. Waadt R, Schmidt LK, Lohse M, Hashimoto K, Bock R, Kudla J: **Multicolor bimolecular fluorescence complementation reveals simultaneous formation of alternative CBL/CIPK complexes in planta.** *Plant Journal* 2008, **56:**505-516.
5. Delventhal R, Rajaraman J, Stefanato FL, Rehman S, Aghnoum R, McGrann GRD, Bolger M, Usadel B, Hedley PE, Boyd L, et al: **A comparative analysis of nonhost resistance across the two Triticeae crop species wheat and barley.** *BMC Plant Biology* 2017, **17:**232.
6. Zimmermann G, Baumlein H, Mock HP, Himmelbach A, Schweizer P: **The multigene family encoding germin-like proteins of barley. Regulation and function in basal host resistance.** *Plant Physiology* 2006, **142:**181-192.
7. Nakagawa T, Kurose T, Hino T, Tanaka K, Kawamukai M, Niwa Y, Toyooka K, Matsuoka K, Jinbo T, Kimura T: **Development of series of gateway binary vectors, pGWBs, for realizing efficient construction of fusion genes for plant transformation.** *J Biosci Bioeng* 2007, **104:**34-41.
