# Supplementary material for: Evolutionarily conserved partial gene duplication in the Triticeae tribe of grasses confers pathogen resistance
Source: Genome Biol. 2018 Aug 15;19:116. doi: 10.1186/s13059-018-1472-7 (PMC6092874; doi:10.1186/s13059-018-1472-7)
Supplement: Supplementary file 3 — Methods S1. Detailed description of materials and methods used in this study. (PDF 183 kb) [file 13059_2018_1472_MOESM3_ESM.pdf]

**Methods S1:**

More detailed description of Materials and Methods.

*Plant and fungal material*

For the TIGS and transient overexpression experiments 7-day-old seedlings of spring barley cv. Maythorpe as well as Golden Promise were used. Seedlings were grown in a plant incubator (Sanyo/Panasonic, Hamburg, Germany) at 20°C constant temperature, 60% rel. humidity and 16 h illumination (intensity level 5) by fluorescent tubes (OSRAM L36W/840). Stable transgenic barley plants were generated in cv. Golden Promise, which represents a γ-mutagenized mutant of cv. Maythorpe. Bombarded leaf segments or transgenic plants were inoculated with Swiss *Bgt* field isolate FAL 92315, or Swiss *Bgh* field isolate CH4.8.

*Transient-induced gene silencing (TIGS)*

Target cDNA sequences for TIGS were selected and analyzed based on sequence-contig information of the HarvEST database, barley 1.83 assembly #35 (http://www.harvest-web.org). Off-target prediction was done using the si-Fi21 software (https://doi.org/10.5447/ipk/2017/9). TIGS constructs were generated and transferred into barley leaf epidermal cells by particle bombardment as described. Leaf segments were inoculated three days after the bombardment with *Bgh* at a density of 140-180 conidia $mm^{-2}$. Transformed GUS-stained epidermal cells as well as haustoria-containing transformed (susceptible) cells were counted 48 h after inoculation, and TIGS effects in Maythorpe were statistically analyzed according to [1]. Statistical analysis in Golden Promise was identical except that the null-hypothetical log(2)-transformed relative SI value did not need any correction for a weak, sequence non-specific RNAi effect that was only observed in Maythorpe.

*Generation and genotyping of transgenic RNAi plants*

The binary RNAi hairpin construct pIPKb009_HvARM1 was generated by using the RNAi-vector pIPKb009 [2]. The LR-reaction with entry clone pIPKTA38_HvARM1 that contained 573 bp of Hv*ARM1* cDNA sequence was carried out as described. This 573 bp RNAi target region of HvARM1 corresponds to 148 bp to 720 bp from start ATG of full-length cDNA clone Hv2142K13 [3]. Immature barley embryos of Golden Promise were transformed with the binary RNAi-vector described above using the *Agrobacterium tumefaciens* strain AGL1 as described [4, 5]. The resulting T0 (primary transformant) plantlets were selected on medium containing hygromycin (50 mg $L^{-1}$).

For segregation analysis in the subsequent generations, individual Hv*ARM1* RNAi plants were tested for hygromycin sensitivity by incubating detached primary leaves in a solution of 200 µg $ml^{-1}$ hygromycin for 7 days. Individuals were also examined by PCR for the presence of the *hptII* selectable marker gene. The presence of the Hv*ARM1* RNAi construct in transgenic plants was examined by PCR with specific primers combinations for *35S* promoter (sense) and *RGA2* intron (antisense), as well as for *RGA2* intron (sense) and *35S* terminator (antisense) (see Additional file 1: Table S9 for primer sequences). Only transformation events containing both inverted repeats and the *hptII* marker gene were selected for further analysis.

*Inoculation and evaluation of transgenic plants*

Phenotypic evaluation of *Bgh* and *Bgt* interactions was done microscopically on second, detached leaves of 12-14 day-old plants placed on phytoagar plates (23,2 cm x 23,2 cm) inoculated at a spore density of 30-40 conidia mm$^{-2}$. Inoculated leaf segment were incubated as described for 48 h (*Bgh*) or 72 h (*Bgt*) followed by staining with Coomassie brilliant blue R 250 [6]. The number of growing colonies/area was counted manually under a standard bright-field microscope at 100 x magnification.

*Reverse transcription-qPCR*

For transcript profiling of Hv*ARM1* and Hv*PUB15* in pathogen-challenged barley cv. Ingrid was used. 7-day-old seedlings grown in pots of 12-14 cm diameter were inoculated at a spore density of 80-100 conidia mm$^{-2}$ of *Bgh* or *Bgt*. Total RNA was isolated from adaxial epidermal peels of primary leaves 6, 12, 24, 48 and 72 h after inoculation using the RNeasy Plant Mini Kit with on-column DNase digestion (Qiagen, Hilden, Germany). Two micrograms of RNA were reverse-transcribed using RevertAid Reverse Transcriptase (Thermo Fisher Scientific, Waltham, MA USA). Amplification and detection of fluorescent signal was performed on a 7900 HT Fast Real-Time PCR system (Life Technologies/Applied Biosystems, Darmstadt, Germany). Transcripts were quantified using TaqMan probes in a reaction volume of 10µL in triplicates using Maxima Probe qPCR Mastermix (Thermo Fisher Scientific, Waltham, MA USA). Thermal cycling conditions consisted of initial denaturation at 95°C for 10 minutes followed by 35 cycles of (95°C/15 s, 58°C/30 s, 72°C/30 s). Ubiquitin conjugating enzyme 2 (Hv*UBC*, Acc. AK252586.1) was used as internal normalization standard. To quantify the transcript levels in each sample, a standard curve for each gene with fourfold serial dilution was made from pooled RNA samples of both non-inoculated and inoculated plants. Three technical replicates were included for each RNA sample. Transcript quantities were determined using the SDS.2.2.2 software (Life Technologies GmbH, Darmstadt, Germany). Statistical significance for differential expression based on absolute transcript quantity was calculated by one-way ANOVA with Bonferroni's multiple comparisons test using GraphPad Prism version 6.0.2 for Windows (GraphPad Software, San Diego, CA).
To determine Hv*ARM1* and Hv*PUB15* transcript levels in transgenic plants ($T_3$), the same procedure was followed except that the total RNA was isolated from the 4$^{th}$ entire leaf and the standard curve was made using RNA from azygous control. Statistical significance of differences between transgenic events and azygous plants are calculated student's t-test using GraphPad Prism. For TaqMan probe sequences and primers, see Additional file 1: Table S9.

*Array-based transcript profiling*

Transcript profiles of a few selected genes were also extracted from an array dataset initially designed to compare host and nonhost interactions in barley and wheat to a range of pathogens including *B. graminis*. Seven-day-old barley plants of cv. Vada were inoculated at a spore density of approximately 50 conidia mm$^{-2}$ of *Bgh* or *Bgt*, and the abaxial epidermis of inoculated primary leaves or from non-inoculated control leaves was peeled 6, 12, 24 and 74 h after inoculation. Total RNA was extracted by using the RNeasy Plant Mini Kit with on-Column DNase digestion (Qiagen, Hilden, Germany), analyzed for quantity and quality by using an Agilent Bioanalyzer 2100 (Agilent Technologies, Santa Clara, CA) and hybridized to a 44K Agilent oligonucleotide custom array of barley as described [7]. Single-channel array processing was utilized followed by data normalization with default parameters, and significant transcript-regulation events were determined by using GeneSpring GX (v11.5.1) software (Agilent). Data are presented as log(2)-transformed, quantile-normalized signal

intensity plot, and statistical significance of differences between inoculated and control samples was calculated by unpaired t-test using GeneSpring without multiple testing correction. Data are based on three independent, serial inoculation experiments.

*Yeast-2-hybrid screening*

A yeast-2-hybrid-(Y2H) screening protocol was performed according to the Yeast Handbook and manual of Matchmaker™ library construction and screening kits (Takara/Clontech Laboratories, Saint-Germain-en-Laye, France). Full length coding sequence of HvARM1 (1-442 AA) was PCR amplified and cloned in-frame into pGBKT7 bait vector at *Nde*I and *Sal*I sites and transformed into Y187 strain of *Saccharomyces cerevisiae*. Y2H prey library was developed as described [8] and the library was used to screen seven Mio. mating events. Transformed yeast cells were screened on synthetic dropout (SD) medium without amino acid Leu and Trp. To select the yeast diploids expressing both the bait and interacting prey protein, stringent SD medium without Leu, Trp, His, and Ade was used. Positive prey clones were identified by PCR amplification and sequencing. Retesting of the interaction was done by co-transformation of respective prey- and HvARM1 bait plasmids in AH109 strain as described by the kit.

For targeted Y2H assays, coding region (1-831AA) of HvPUB15 was PCR amplified with *Pfu* polymerase from a full length cDNA clone obtained from NIAS, Japan (http://barleyflc.dna.affrc.go.jp) and sub-cloned in-frame into pGBKT7 vector at *Nde*I and *Swa*I site. HvPUB15 bait construct was co-transformed in AH109 strain with respective positive prey clones of the HvARM1 screening to test for interaction. Yeast spotting in the figure was done as described [9].

*Bimolecular fluorescence complementation*

For BiFC based protein-protein interaction studies using *Agrobacterium tumefaciens*-mediated infiltration of *N. benthamiana* leaves, a GATEWAY compatible-BiFC binary vector set derived from the 35S::$^{GW}$sCFP3A-pBar vector as described [10] was generated. To this end, the N-terminus (aa 1-155) and C-terminus (aa 156-239) of the enhanced yellow fluorescent protein (Venus) were amplified by using the following primer pairs: Spe-c-myc-VenN-fwdTACTAGTATGGAGCAAAAG-TTGATTTCTGAGGAGGATCTTATGGTGAGCAAGGGCGAG -3´ plus XbaI-VenN-rev 5´-CTCTAGACTACTCGATGTTGTGGCG -3´, and SpeI-HA-VenC-fwd 5´-TACTAGTATGTACCCATACGATGTTCCAGATTACGCTGACAAGCAGAAGAACGGCAT-3´ plus XbaI-VenC-rev5´-CTCTAGATTACTTGTACAGCTCGTC-3´. The coding sequence of sCFP3A was previously excised/removed from 35S::$^{GW}$sCFP3A-pBar by restriction with SpeI and XbaI and both Venus fragments were ligated into the linearized vector backbone to generate 35S::$^{GW}$VYNE-pBar and 35S::$^{GW}$VYCE-pBar. Both vectors allow for C-terminal protein fusions to the respective Venus fragment by using GATEWAY cloning technology. Into these vectors were cloned: the wild-type, full-length sequences of HvTHF1, HvClpS1, the wild-type full-length sequences of *HvPUB15* or *HvARM1*, U-box mutants of *HvPUB15*, the ARM-domain (351 to 831 AA) only of *HvPUB15*, or *HvThf1* without N-terminal plastid import signal (-SP). Gateway LR reactions were performed using attL-site adapter-primer PCR fragments (Additional file 1: Table S9). Final constructs were sequence-verified and transformed separately into *A. tumefaciens* strain EHA 105. 48 hours after combined infiltration of the different gene fusion constructs into *Nicotiana benthamiana*, specific fluorescence signals were monitored by using Zeiss LSM 780 confocal laser scanning microscope. Fluorophore signals, chlorophyll autofluorescence and bright field images were

scanned sequentially in channel mode to prevent any crosstalk between fluorescence channels.
*Nicotiana benthamiana* plants were grown under 16 h of light at 22°C day and 20°C night temperature. BiFC constructs were introduced into *Agrobacterium tumefaciens* strain EHA105. For co-infiltration, transformed cells were cultured in YEB medium (0.5% beef extract, 0.1% yeast extract, 0.5% sucrose, and 2mM MgSO4·7$H_2O$) at 28°C overnight. Cells were collected and resuspended in 2x infiltration buffers (10% sucrose, 20 mM glucose, pH 5.6). Equal volume of respective BiFC combinations with a final OD600 of 0.5 each were mixed together and infiltrated into abaxial leaves surface of 4-5 weeks old *N. benthamiana* using a syringe with no needle. Leaf sections were observed under a LSM 780 confocal microscope (Zeiss) 48 h after infiltration. YFP fluorescence was visualized using a 514 nm laser excitation in combination with a 517–560 nm bandpass using ZEN (ver.2.1) software.

*Subcellular localization of fluorescent proteins*
For subcellular localization, coding-sequence of Hv*PUB15* (1-831AA), Hv*ARM1* (1-442) *HvThf1* (1-294 AA) and *HvClpS1* (1-161 AA) were PCR amplified and corresponding PCR products were N- and C-terminally fused in-frame to YFP in pIPKTA48 and pIPKTA49 vectors (Additional file 1: Figure S11 and S12) via Gateway BP and LR reactions (Life Technologies/Invitrogen, Darmstadt, Germany). Resulting YFP-fusion constructs were transiently expressed in 7-day-old barley leaf segments by particle bombardment and examined after 12-24 h of incubation without *B. graminis* inoculation. YFP fluorescence was first detected by using a Zeiss Axio Imager.Z1m microscope, and constructs producing strong fluorescent signals were further analysed by a LSM 780 confocal microscope (Zeiss) using 488 nm excitation in combination with a 505-550 nm bandpass filter.
For testing co-localization of YFP signals within epidermal leucoplasts, the HvThf1- or Hv*ClpS*-YFP-fusion constructs were co-bombarded with plasmid pt-rkCD3-999 containing the plastid marker Rubisco small subunit fused to red fluorescing mCherry [11]. Recordings of YFP and mCherry (514 nm excitation, 590-644 nm bandpass filter) were taken by CLSM 24 h after bombardment.

*Exome capture sequencing*
Library preparation and sequencing: Genomic DNA (gDNA) was extracted from barley leaf material from a single plant for each accession. After integrity check, quantification and normalization to 20 ng/ul the gDNA was fragmented to a size range of 180-200 bp and whole genome libraries were prepared according to the Kapa Library Preparation protocol. Libraries were quantified and analyzed electrophoretically. Libraries were pooled in 8-plex and used for the hybridization with the barley SeqCap Ez oligo pool (Design Name: 120426_Barley_BEC_D04, [12]. Capture beads were used to pull down the complex of capture oligos and genomic DNA fragments. Enriched fragments were amplified by PCR and the final library was quantified by qPCR and visualized by Agilent Tape Station. Sequencing libraries were used for cluster amplification on the cBot. The clustered flow cells were sequenced on Illumina HiSeq2000 with a 100 bp paired-end run module.
Sequence processing and alignment: Sequence quality control was assessed with FastQC (Babraham Institute: http://www.bioinformatics.babraham.ac.uk/projects/fastqc/). Raw Illumina reads were then quality trimmed to a base quality of 20 from both ends with Trimmomatic version 0.30 [13]. Only correctly paired reads longer than 36 bp were used for further processing. Trimmed reads were then mapped to the reference genome (http://webblast.ipk-gatersleben.de/barley_ibsc/downloads/) with BWA version 0.7.5a using

the mem algorithm with default parameters [14] and retaining only properly paired reads. A total number of approximately 24 million reads were mapped to the reference genome.

SNP calling and validation: Variant calling and realignment around indels were performed with GATK, version 2.7.4 (https://www.broadinstitute.org/gatk/). All the final BAM files were processed together using GATK UnifiedGenotyper with default parameters and minimum base quality of 30. The raw variant calls produced were initially hard filtered by requiring QD > 30.0, MQ > 40.0 and sample DP >= 10. Further validation steps based on already known SNPs were carried out and finally more than 64.5M variants were selected from 403 accessions. These were further filtered imposing 80% of samples being represented at each SNP locus, and a dataset of more than 2M SNPs was produced, suitable for allele mining for the two genes under investigation (full information about genome wide variants from this dataset will be published elsewhere).

**References cited in Supplementary Information only.**


1. Douchkov D, Lück S, Johrde A, Nowara D, Himmelbach A, Rajaraman J, Stein N, Sharma R, Kilian B, Schweizer P: **Discovery of genes affecting resistance of barley to adapted and non-adapted powdery mildew fungi.** *Genome Biol* 2014, **15:**518.
2. Himmelbach A, Zierold U, Hensel G, Riechen J, Douchkov D, Schweizer P, Kumlehn J: **A set of modular binary vectors for transformation of cereals.** *Plant Physiology* 2007, **145:**1192-1200.
3. Matsumoto T, Tanaka T, Sakai H, Amano N, Kanamori H, Kurita K, Kikuta A, Kamiya K, Yamamoto M, Ikawa H, et al: **Comprehensive sequence analysis of 24,783 barley full-length cDNAs derived from 12 clone libraries.** *Plant Physiol* 2011, **156:**20-28.
4. Schultheiss H, Hensel G, Imani J, Broeders S, Sonnewald U, Kogel KH, Kumlehn J, Hückelhoven R: **Ectopic expression of constitutively activated RACB in barley enhances susceptibility to powdery mildew and abiotic stress.** *Plant Physiology* 2005, **139:**353-362.
5. Hensel G, Valkov V, Middlefell-Williams J, Kumlehn J: **Efficient generation of transgenic barley: the way forward to modulate plant-microbe interactions.** *J Plant Physiol* 2008, **165:**71-82.
6. Schweizer P, Gees R, Mosinger E: **Effect of Jasmonic Acid on the Interaction of Barley (Hordeum-Vulgare L) with the Powdery Mildew Erysiphe-Graminis F Sp Hordei.** *Plant Physiology* 1993, **102:**503-511.
7. Chen XW, Hedley PE, Morris J, Liu H, Niks RE, Waugh R: **Combining genetical genomics and bulked segregant analysis-based differential expression: an approach to gene localization.** *Theoretical and Applied Genetics* 2011, **122:**1375-1383.
8. Hoefle C, Huesmann C, Schultheiss H, Bornke F, Hensel G, Kumlehn J, Hückelhoven R: **A barley ROP GTPase ACTIVATING PROTEIN associates with microtubules and regulates entry of the barley powdery mildew fungus into leaf epidermal cells.** *Plant Cell* 2011, **23:**2422-2439.
9. Maple J, Moeller SG: **Yeast Two-Hybrid Screening.** In *Methods in Molecular Biology. Volume* 362. Edited by Rosato E. Totowa, NJ: Humana Press Inc.; 2007: 207-223
10. Thormahlen I, Meitzel T, Groysman J, Ochsner AB, von Roepenack-Lahaye E, Naranjo B, Cejudo FJ, Geigenberger P: **Thioredoxin f1 and NADPH-Dependent Thioredoxin Reductase C Have Overlapping Functions in Regulating Photosynthetic Metabolism and Plant Growth in Response to Varying Light Conditions.** *Plant Physiol* 2015, **169:**1766-1786.

11. Nelson BK, Cai X, Nebenfuhr A: **A multicolored set of in vivo organelle markers for co-localization studies in Arabidopsis and other plants.** *Plant Journal* 2007, **51:**1126-1136.
12. Mascher M, Richmond TA, Gerhardt DJ, Himmelbach A, Clissold L, Sampath D, Ayling S, Steuernagel B, Pfeifer M, D'Ascenzo M, et al: **Barley whole exome capture: a tool for genomic research in the genus Hordeum and beyond.** *Plant J* 2013, **76:**494-505.
13. Bolger AM, Lohse M, Usadel B: **Trimmomatic: a flexible trimmer for Illumina sequence data.** *Bioinformatics* 2014, **30:**2114-2120.
14. Li H, Handsaker B, Wysoker A, Fennell T, Ruan J, Homer N, Marth G, Abecasis G, Durbin R, Genome Project Data Processing S: **The Sequence Alignment/Map format and SAMtools.** *Bioinformatics* 2009, **25:**2078-2079.
